# Judgments in the Sharing Economy: The Effect of User-Generated Trust and Reputation Information on Decision-Making Accuracy and Bias

**DOI:** 10.3389/fpsyg.2021.776999

**Published:** 2021-11-16

**Authors:** Mircea Zloteanu, Nigel Harvey, David Tuckett, Giacomo Livan

**Affiliations:** ^1^Department of Criminology and Sociology, Kingston University London, Kingston, United Kingdom; ^2^Department of Psychology, Kingston University London, Kingston, United Kingdom; ^3^Department of Experimental Psychology, University College London, London, United Kingdom; ^4^Centre for the Study of Decision-Making Uncertainty, University College London, London, United Kingdom; ^5^Department of Computer Science, University College London, London, United Kingdom; ^6^Systemic Risk Centre, London School of Economics and Political Sciences, London, United Kingdom

**Keywords:** accuracy, bias, digital identity, reputation, sharing economy, trustworthiness, user judgment, user-generated content

## Abstract

The growing ecosystem of peer-to-peer enterprise – the Sharing Economy (SE) – has brought with it a substantial change in how we access and provide goods and services. Within the SE, individuals make decisions based mainly on user-generated trust and reputation information (TRI). Recent research indicates that the use of such information tends to produce a positivity bias in the perceived trustworthiness of fellow users. Across two experimental studies performed on an artificial SE accommodation platform, we test whether users’ judgments can be accurate when presented with diagnostic information relating to the quality of the profiles they see or if these overly positive perceptions persist. In study 1, we find that users are quite accurate overall (70%) at determining the quality of a profile, both when presented with full profiles or with profiles where they selected three TRI elements they considered useful for their decision-making. However, users tended to exhibit an “upward quality bias” when making errors. In study 2, we leveraged patterns of frequently vs. infrequently selected TRI elements to understand whether users have insights into which are more diagnostic and find that presenting frequently selected TRI elements improved users’ accuracy. Overall, our studies demonstrate that – positivity bias notwithstanding – users can be remarkably accurate in their online SE judgments.

## Introduction

The Sharing Economy (SE) is a multifaceted and complex concept that can be difficult to define (Frenken and Schor, 2017). Within the current scope, it is described as an ecosystem of online platforms focused on the temporary exchange of goods and services through peer-to-peer (P2P) connections (Botsman and Rogers, 2010; Fraiberger and Sundararajan, 2017; Teubner and Hawlitschek, 2018). The SE is considered to be a socio-economic movement with immense potential for positive economic growth (Dabbous and Tarhini, 2021). For a more comprehensive overview of the multiple aspects of the SE, see Eckhardt et al. (2019) and Hossain (2020).

Given the nature of the SE, P2P interactions can be complex, uncertain, and risky (Luo et al., 2021). Typically, users must rely on the information presented on such platforms to make decisions, yet this is predominantly user-generated content (UGC) relating to trust and reputation information (TRI). Importantly, the diagnosticity and reliability of such information are often difficult to assess, and little is known about how users perceive and utilize such information to make decisions. From the existing research, we know that users are less critical on SE platforms than in other online environments (Zervas et al., 2021) and tend to be overly positive in their perceptions and judgments of other users (Zloteanu et al., 2018).

The current research is organized in the following way. First, we provide a *Theoretical Framework*, which overviews relevant literature. Second, the *Present Research* section details our research questions and their operationalization. Third, two experimental studies are presented, describing their hypotheses, methodology, results, and interim discussions. In the *Conclusion* section, we provide a general discussion incorporating the findings and consider the theoretical and practical implications of the research, including limitations and future directions.

## Theoretical Framework

### Trust and Reputation

The function of trust and reputation have received significant attention in the SE literature, due to their central role in how such marketplaces operate (Wang and Emurian, 2005; Hawlitschek et al., 2016). SE platforms rely heavily on TRI to function and proliferate (Jiang and Lau, 2021), actively encouraging and providing users with multiple methods of communicating such information to peers (Resnick and Zeckhauser, 2002), such as providing ratings, commenting, verifying one’s identity and credentials, uploading images, and so on with the aim of building a perception of trustworthiness and a positive reputation toward the platform and its users (Teubner, 2014; Ert et al., 2016). The role of such mechanisms is to reduce the implicit uncertainty of interactions on the platform, the assumed risk, and to promote commerce (Mayer et al., 1995; Flavián et al., 2006; Sparks and Browning, 2011). This is crucial, as information asymmetry, inaccurate descriptions, and unmet expectations can have strong negative impacts on user engagement with the SE (Ding et al., 2021). In turn, users’ ability to assess the trustworthiness of other users, especially those with whom they wish to interact, is fundamental to the operation of an SE platform. SE users seek TRI about other users when given the chance (Chatterjee et al., 2001; Zloteanu et al., 2018), and such information has been shown to impact behavior (Casaló et al., 2011; Zervas et al., 2021).

Alongside trust metrics, SE platforms also foster reputation building, both of their userbase and of the platform itself. Reputation here reflects the broad perception of the credibility and integrity of the information on the platform, the safety and professionalism of its services, and the community itself. Unlike trust, reputation metrics tend to feature aggregate sentiments regarding either specific users, services, or the platform (e.g., a score out of 5, star ratings, or number of services rendered) (Slee, 2017). It has been argued that an SE platform’s reputation can act in lieu of more objective regulatory mechanisms, with a positive reputation providing similar benefits to established credibility services typically found in non-SE marketplaces (Green, 2012; Yacouel and Fleischer, 2012). Thus, both trust and reputation are viewed by experts as valuable commodities and central to the successful operation of the SE.

However, as most TRI is UGC there is an implicit uncertainty as to its authenticity and diagnosticity, especially for user decision-making. Indeed, research shows that the majority of TRI reflects a hyper-positive view (Zervas et al., 2021), where the majority of reviews, comments, or ratings are much more positive than would be expected by chance or when compared to non-SE e-commerce platforms (McKnight et al., 2002a, b). This underreporting of negative TRI may be in part due to users fearing retaliation or ostracism if they provide critical feedback (Bolton et al., 2013; Zervas et al., 2021), resulting in what is referred to as *tainted prosociality* (Zaki et al., 2021). However, due to the asymmetry in information between consumers and prosumers inherent to SE platforms (e.g., anonymity, lack of accountability), users are forced to rely on such TRI to inform their decision-making. Yet, how users use such information, and the effects different TRI elements have on judgment is still relatively unknown.

### User Judgment and Decision-Making

Decision-making in SE environments can be a complex task. In addition to the aforementioned hyper-positivity of information, calling into question its authenticity and diagnosticity, users must integrate various cues into their decision-making, each of varying quality, type, and potential usefulness.

The decision-making literature tells us that people are cognitive misers who typically struggle to incorporate large amounts of diverse information from multiple sources into their judgments (Tversky and Kahneman, 1974; Fiske and Taylor, 2013). Instead of employing complex decision-making strategies, people use quick, but efficient, heuristics, referred to as fast-and-frugal approaches (Gigerenzer and Gaissmaier, 2011). People are also poor at describing their decision-making strategies or the factors that they incorporate into their judgments (Harries and Harvey, 2000). Notably, while it was initially believed that the use of such heuristics results in poorer decision-making (Piatelli-Palmarini, 1994), it is now acknowledged to provide comparatively accurate judgments (Gigerenzer and Gaissmaier, 2011). These patterns of reasoning seem to also extend to SE environments, as users assume they can incorporate multiple cues into their judgments or *a priori* believe that specific factors affect their involvement with the SE environment when this does not reflect real-world behavior (e.g., privacy; Martínez-Navalón et al., 2021). Furthermore, users even request additional information or indicate strong preferences for specific sources, yet seem to be unable to incorporate more than around three TRI elements to reach a stable decision (Zloteanu et al., 2018; see, also Harries and Harries, 2001). Although users tend to have strong preferences for specific TRI elements, this is attributed to a form of arbitrary coherence (Nisbett and Wilson, 1977; Ariely et al., 2003) – developing preferences in the absence of specific criteria, due to repeated exposure to and/or the expectation of such information being present – without any empirical evidence demonstrating that their preferences reflect a rational and optimal strategy. Thus, the question remains: can users be accurate in their SE judgments if presented with diagnostic TRI?

Notably, most research on user behavior in the SE has focused on real-world data, where an objective understanding of the potential for accurate judgment is difficult to obtain. The hyper-positive culture in ratings and interactions – encompassed by the so-called “5-for-5” practice of exchanging 5-star ratings – severely reduces the diagnosticity of existing information (Livan et al., 2017). Relying on naturalistic data can restrict researchers’ ability to uncover the mechanisms by which users make their decisions and understand how they incorporate TRI into their judgments. As such, an experimental approach is proposed, allowing researchers to measure user behavior in a controlled environment, restricting confounding variables, and permitting for causal links to be discovered.

## Present Research

The current research is an extension of the work by Zloteanu et al. (2018) relating to the influence of TRI on user perception and judgment. In our previous work, we found that users are equally influenced by various TRI elements, display strong preferences for specific information, but seem to reach a stable perceptual and judgment pattern after incorporating around three TRI elements, regardless of their content. A natural extension of that work is to consider if users can be accurate in their SE judgments when provided with diagnostic TRI. Namely, while the research on real-world data shows overly positive and undiagnostic TRI on SE platforms (Zervas et al., 2021) and that users tend to be overly positive in their perceptions of other users (Zloteanu et al., 2018), the question remains whether such effects are simply due to the ecosystem itself or a lack of ability by users to accurately interpret and integrate TRI into their judgments. Thus, using an experimental approach, if we provide an SE platform where TRI is diagnostic of the quality of the goods or services offered, can users perceive and incorporate this information to make accurate judgments? This question is pertinent given the mechanism promoted by SE platforms. If users are inaccurate in their judgments, this will provide evidence for the potential risks associated with participation in such an ecosystem (i.e., a false impression of informed decision-making). Alternatively, if users are accurate, then it highlights the importance of ensuring that the information present on such platforms is authentic and informative (i.e., promote mechanisms for the dissemination of diagnostic TRI).

To our knowledge, this is the first experimental approach to investigating user accuracy in SE environments. Our aim is to provide clear and casual insight into the role TRI has on user judgment and perceptions of hosts, uncovering how different elements influence decision-making. We also provide a new methodology for such investigations and discuss future research avenues. The findings from our research are relevant to informing the governing of future SE platforms, especially relating to platform managers and policymakers, moving them toward offering beneficial information and promoting positive user behavior and communities. Given the importance in recent years of privacy and security, providing a greater understanding of how people make decisions on SE platforms is highly relevant to the future of the ecosystem (see Ribeiro-Navarrete et al., 2021).

The central research questions we address relate to users’ (i.e., guests) ability to judge and integrate the various TRI elements present on accommodation-based SE platforms when forming judgments relating to quality and provider (i.e., hosts) characteristics. We approach this problem by considering the decision-making literature and research on human cognition (Gigerenzer and Gaissmaier, 2011) and knowledge of how SE platforms operate (Zloteanu et al., 2018; Zervas et al., 2021). Our approach considers (1) how accurate users are when provided with diagnostic TRI, and (2) if they demonstrate specific judgment response patterns when presented with uncertain information which align with their pre-existing beliefs (i.e., a positivity bias).

## Study 1: Research Questions and Hypotheses

Three types of accommodation profiles were created where the quality of the TRI varied, suggestive of the quality of good, mixed, or bad profiles. Here, we considered how accurate users’ perception of online SE accommodation-based profiles can be when presented with either full TRI about potential hosts and their properties or given the option to select three elements that they consider most useful. Our hypotheses were as follows:

**H_1_**: Users in the Visible condition (i.e., where complete information is visible and detailed through seven distinct TRI elements) will be more accurate at determining the quality of rooms than users in the Reveal condition (i.e., where users are free to select which three TRI elements to see).
**H_2_**: Users will be more positive toward hosts in the Visible condition (as measured by perceived sociability, credibility, and trustworthiness).
**H_3_**: Users in the Reveal condition will be more confident in their decisions, owning to the fact that they consider they are making the most informed judgments for each profile.
**H_4_**: Judgments will differ between the three profile conditions (Good, Mixed, and Bad).

### Method

**FIGURE 1:**
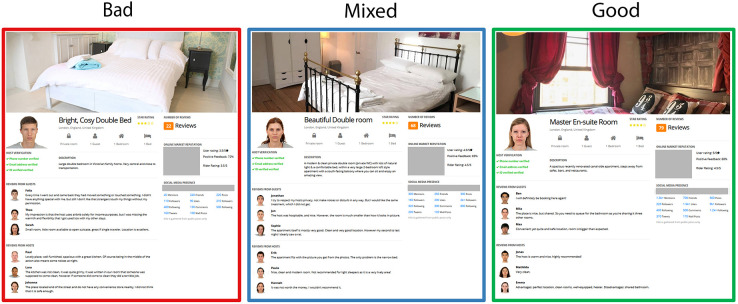
Example of a profile generated in the Bad, Mixed, and Good condition. Commercial logos have been removed for publication.

In both this study and the following one, we adopt an experimental methodology. This was because it is the only empirical approach that provides the level of control needed to produce decisive tests of our hypotheses. Although experimental approaches are occasionally criticized as lacking in external validity, that issue is not salient here. We simulated a typical SE site with a high degree of fidelity (Figure 1) and users interacted with the site via their own computer systems just as they would when dealing with real online platforms.

#### Participants and Design

A mixed design was employed, with Condition (Visible or Reveal) as the between-participants factor, and Profile (Good, Mixed, and Bad) as the within-participants factor. There were seven dependent measures of interest: profile quality, rent decision, judgment confidence, perceived sociability of the host, perceived credibility of the host, perceived trustworthiness of the host, classification accuracy, and judgment bias.

A total of 116 participants (63 males, 53 females; *M_*Age*_* = 35.28, *SD* = 9.92; Visible condition *n* = 59, Reveal condition *n* = 57) were recruited through Amazon’s Mechanical Turk (mTurk) in exchange for a flat fee of $1.00. A sensitivity analysis for a 2 (Condition: Visible or Reveal) x 3 (Profile: Good, Mixed, and Bad) factorial interaction set at 80% power and alpha of 0.05 (two-sided), estimates that effect sizes as small as Cohen’s *f* = 0.21 o r $\eta_{P}^{2}$ = 0.04 can be reliably detected with the current sample size. Informed consent was obtained from all participants. All current procedures have been reviewed and received approval from the University College London Research Ethics Committee (CEHP/2015/534).

#### Materials and Stimuli

The current approach used an adapted design from Zloteanu et al. (2018) for the artificial SE platform. Three types of SE accommodation-type profiles were generated using our artificial platform (inside Gorilla’s experiment builder^1^). For details on the construction of this platform, see Zloteanu et al. (2018).

All profiles (*N_*S*__*timuli*_* = 30) were randomly generated on a per participant basis (using predetermined parameters for each condition) and contained elements generally seen on accommodation-based platforms (e.g., a photo of the advertised room, a description of the room, a photo of the host), alongside seven user-generated TRI elements (see Supplementary Materials). The profiles were of private rooms in the host’s house which they were renting to potential guests. The seven TRI elements of interest were: star ratings, number of reviews, 3x comments from guests, 3x comments from other hosts on the platform, online market reputation of the host, social media presence of the host, and host verification status. As in Zloteanu et al. (2018), multiple factors were controlled in the creation of the profiles to ensure ecological validity (e.g., the host profile photos were match-controlled for similar ratings of perceived facial trustworthiness, attractiveness, and dominance; Ma et al., 2015). However, here, the quality of the profiles was manipulated through the seven elements to reflect either a good, mixed, or bad TRI.

The quality manipulation was achieved by varying the information contained in each TRI element. In the Good condition, all comments available (guests and hosts) were positive (e.g., “Beautiful, spacious and very clean apartment with an excellent and friendly host”), the host verification was fully confirmed (3/3 details), had a high star rating (e.g., 4.5/5 stars), had a high number of reviews (e.g., 85) indicating many previous individuals selected the location, and had very high online market reputation and social media presence (see Supplementary Materials for details on the generation process and variance parameters for each element). In the Mixed condition, comments (guests and hosts) were more neutral (e.g., “Basic crashpad that did the job of needing a place at short notice”), while the other elements had lower ratings compared to the profiles in the Good condition (e.g., 3.5 stars, 50 total reviews, weaker online presence). In the Bad condition, comments were negative overall (e.g., “No privacy at all. The bathroom and shower were very dirty and in poor condition”), host verification was poor (e.g., 1/3), and the profile had a poor star rating (e.g., 2.5/5) and a low number of overall reviews (e.g., 4), as well as a very poor online and social media presence (e.g., 80 likes, 150 followers). For an example of each profile type, see Figure 1.

The profiles in the Reveal condition differed from their Visible counterparts as the seven TRI elements were obscured at the start of each trial (displayed as gray boxes with a token logo on each). Users would click on the three elements they wanted to view. The platform forced users to spend all tokens before proceeding to the next trial, and their selection was final with no option to refund tokens and select again.

#### Procedure

Each participant was randomly allocated into either the Visible or Reveal condition. The instructions and layout of the experiment were kept as consistent as possible between the two conditions, with the only difference of note being that in the Reveal condition participants were provided three tokens per profile to spend on revealing any of the seven obscured TRI elements (i.e., star rating, number of reviews, host verification, online market reputation, social media reputation, guest reviews, host reviews) to assist in their decision-making.

Before the main task began, participants were provided with instructions on what the task entailed, and what their responses should be. An initial example profile (with placeholder information) was presented to allow them to explore the TRI elements and the reveal process if assigned to the Reveal condition. This was followed by six practice profiles – two good, two mixed, and two bad – presented in random order, which provided feedback regarding the decision participants made (i.e., judging the profile as either “good,” “mixed,” or “bad”) after each profile. The feedback included information on if they were correct or incorrect and, if incorrect what their answer should have been (e.g., if they selected “mixed” for a good profile, the text read as “Ouch, looks like you’re wrong this time! This profile is Good, not Mixed!”. At the end of the six trials, they were given a percentage (%) accuracy score for the practice block.

The 30 profiles (10 Good, 10 Mixed, 10 Bad) were randomly presented to participants. After each profile, participants were asked to provide an evaluation of the profile quality, phrased as “Was this a good, mixed or a bad profile?” with a forced-choice ternary response (“Good,” “Mixed,” or “Bad”), a forced-choice binary rent decision (“Yes” or “No”), a confidence judgment (10-point Likert-type scale, anchored in 1 = Not at all confident to 10 = Very confident), followed by three questions relating to the perceived characteristics of the host. These were perceived sociability phrased as “How sociable do you think the host would be?” (10-point Likert-type scale; 1 = Not at all Sociable to 10 = Very Sociable), perceived trustworthiness phrased as “How would you rate the trustworthiness of the host?” (10-point Likert-type scale; 1 = Very Untrustworthy to 10 = Very Trustworthy), and information credibility phrased as “How credible do you think the information about this room is?” (10-point Likert-type scale; 1 = Not at all Credible to 10 = Very Credible).

In the Reveal condition, participants had the additional instruction of spending three tokens per profile to reveal TRI elements that they believed would assist them in deciding the profile’s quality. All seven elements were obscured at the start of the trial and were revealed only once the users clicked the element. After all tokens were spent no additional elements could be revealed, nor could the existing elements be obscured again. As this was done on a per profile basis, users could select any combination of three elements every time. The platform forced users to spend all tokens before being able to progress to the next profile. To ensure that participants attended to the information on screen, after each trial they were asked a question regarding the content of the profile they had just seen (e.g., “Was the room modern or traditional/rustic?”) with an answer field that allowed them to type out their response. The task took on average 25 minutes to complete. Participants were fully debriefed at the end.

#### Data Analysis

All analyses were conducted in the R environment (R Core Team, 2020). To test our experimental hypotheses, we analyzed participants’ data on all relevant measures to uncover behavioral differences in judgment and perception between the experimental conditions and profile types. Bayesian mixed-effects models were constructed using the *brms* package (Bürkner, 2018), posterior inferences were estimated using the *bayestestR* package (Makowski et al., 2019b), and marginal effects were plotted using the *ggeffects* package (Lüdecke, 2018). To ensure stable estimates, especially for per parameter Bayes Factors (BF), models were fitted over 40,000 iterations, using weakly informative priors (verified for model descriptive adequacy using prior predictive checks). The use of weakly (non-uniform) informative priors is recommended when *a priori* assumptions of the effect distributions for interactions are not available or possible. However, it should be noted that this biases all BFs to favor the null hypothesis over the alternative. For each model, we provide the coefficient estimate (β) as the median value of the posterior distribution, 89% credible intervals (Highest Density Interval [HDI]), a per parameter probability of direction (PD), a full region of practical equivalence (% in ROPE) indices which provide more reliable inferences relating to accepting or rejecting the null (Wagenmakers et al., 2010), the Brooks-Gelman-Rubin scale reduction factor (*R̂*), and a BF (Savage-Dickey density ratio) calculated as evidence for H_*a*_ relative to the H_0_. Model convergence and reliability were assessed using posterior predictive checks (PPC) and visual inspection of the parameter space. All models had Condition and Profile as fixed effects and participant as a random effect. As each profile was generated ad lib, trial was not included as a random effect as this would not provide any benefit for variance control.

### Results

User responses to all dependent variables were analyzed considering the two experimental groups (Reveal and Visible) and the three profile conditions (Good, Mixed, and Bad). Data from 10 participants had missing values for the classification decision, rent decision, and judgment confidence. Therefore, for those specific analyses (i.e., rent decision, accuracy, bias, and confidence) the data were modeled on *N* = 106 participants (59 males, 47 females; *M_*Age*_* = 35.42, *SD* = 10.11; Visible condition *n* = 51, Reveal condition *n* = 55).

#### Decision to Rent

A Bayesian logistic mixed-effects model was conducted (using a Bernoulli likelihood), considering the effects of Condition (Reveal or Visible) and Profile (Good, Mixed, and Bad) on rent decisions. The model results are displayed in Table 1.

**TABLE 1:** Summary of the model fitted to the rent data, considering the experimental condition (Visible or Reveal) and Profile type (Good, Mixed, and Bad).

	β	89% HDI	PD	% in ROPE	*R̂*	BF_10_
Intercept	0.05	[−0.08, 0.18]	72.98	94.34	1.00	0.99
Condition [Visible]	–0.03	[−0.17, 0.11]	64.82	94.25	1.00	0.96
Profile [Good]	–0.95	[−1.05, −0.85]	100.00	0.00	1.00	>999
Profile [Mixed]	–1.44	[−1.54, −1.34]	100.00	0.00	1.00	>999
Condition [Visible]: Profile [Good]	0.02	[−0.10, 0.13]	59.12	98.48	1.00	0.75
Condition [Visible]: Profile [Mixed]	0.17	[0.05, 0.29]	98.88	56.05	1.00	10.17

*Results are given on the logit (not the response) scale.*

**FIGURE 2:**
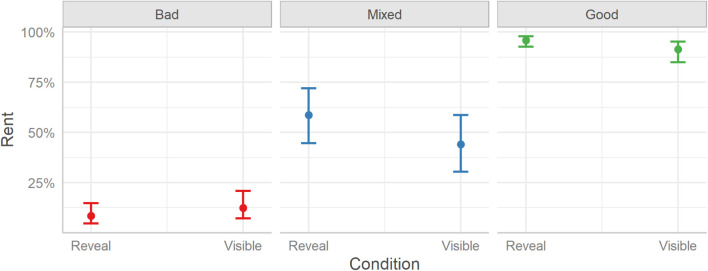
The probabilities of deciding to rent a room between the two experimental conditions (Reveal and Visible) split over the three Profile conditions (Bad, Mixed, and Good). Error bars indicate 95% credible intervals.

The model suggests that the experimental condition (Reveal or Visible) did not have an impact on rent decisions overall. Thus, users made similar rent decisions toward the three types of profiles regardless of whether they saw full profiles (Visible) or profiles containing only three user-selected TRI elements (Reveal). See Figure 2.

Considering the differences between the three profile conditions, the model finds that rent decisions differed between all conditions. Users decided to rent fewer Bad rooms (23%) than Good rooms (80%), Median [Mdn] = −4.92, HDI_89__%_ [−5.23, −4.65], BF_10_ > 999, Odds Ratio [OR] = 0.09, or Mixed rooms (51%), Mdn = −2.22, HDI_89__%_ [−2.44, −1.99], BF_10_ > 999, OR = 0.34. Similarly, users decided to rent more Good rooms than Mixed rooms, Mdn = 2.70, HDI_89__%_ [2.48, 2.94], BF_10_ > 999, OR = 3.83.

The Condition X Profile interaction was also considered; however, it should be noted that although the 89% HDI falls outside 0, the interval is wide and, as 56.05% of the posterior distribution falls within the ROPE, this suggests that any effect is highly uncertain and may be equivalent to zero (see Rouder et al., 2018). Indeed, *post hoc* contrasts did not reveal any differences in rent decisions between the two conditions (Reveal or Visible) in either the Bad (Mdn = −0.15, HDI_89__%_ [−0.40, 0.09], BF_10_ = 1.35, OR = 0.86), Mixed (Mdn = 0.13, HDI_89__%_ [−0.11, 0.37], BF_10_ = 1.20, OR = 1.47), or Good (Mdn = 0.16, HDI_89__%_ [−0.08, 0.41], BF_10_ = 1.48, OR = 1.14) profile condition.

#### Accuracy

A Bayesian logistic mixed-effects model was conducted (using a Bernoulli likelihood), considering the effects of Condition (Reveal or Visible) and Profile (Good, Mixed, and Bad) on user accuracy. Here, accuracy reflected the users’ ability to correctly classify the profiles they saw as either good, mixed, or bad. If a user’s answer matched the type of profile (e.g., a Good profile was classified as “good”) it would be taken as correct = 1; if not it would be taken as incorrect = 0. The model results are displayed in Table 2.

**TABLE 2:** Summary of the model fitted to the accuracy data, considering the experimental condition (Visible or Reveal) and Profile type (Good, Mixed, and Bad).

	β	89% HDI	PD	% in ROPE	*R̂*	BF_10_
Intercept	0.92	[0.77, 1.08]	100.00	0.00	1.00	>999
Condition [Visible]	–0.01	[−0.23, 0.21]	53.19	81.25	1.00	0.03
Profile [Good]	–0.95	[−1.07, −0.83]	100.00	0.00	1.00	>999
Profile [Mixed]	–0.48	[−0.59, −0.37]	100.00	0.00	1.00	>999
Condition [Visible]: Profile [Good]	0.20	[0.03, 0.37]	96.96	43.67	1.00	0.12
Condition [Visible]: Profile [Mixed]	0.07	[0.08, 0.23]	77.99	85.91	1.00	0.03

*Results are given on the logit (not the response) scale.*

**FIGURE 3:**
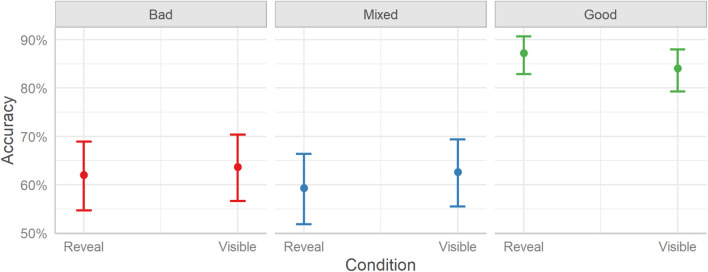
User accuracy in categorizing a profile as bad, mixed, or good in the two experimental conditions (Reveal and Visible) split over the three Profile conditions (Bad, Mixed, and Good). Error bars indicate 95% credible intervals.

The model shows that, like the rent decisions, seeing either the full profiles (Visible) or partial profiles (Reveal) had similar overall effects on accuracy, β = −0.01, HDI_89__%_ [−0.23, 0.21], ROPE% = 81.25, BF_10_ = 0.03. Similarly, no interaction between condition and profile proved significant (PDs ≤ 96.96).^2^ To investigate the differences in accuracy between the profile conditions, we plotted the marginal effects; see Figure 3.

The estimated user accuracy for Bad profiles is 63%, for Mixed profiles is 61%, and for Good profiles is 86%. Considering the differences between profile types, Bad profiles were identified with lower accuracy than Good profiles (Mdn = −1.27, HDI_89__%_ [−1.43, −1.09], BF_10_ > 999, OR = 0.28), but Mixed profiles were rated with similar accuracy (Mdn = 0.08, HDI_89__%_ [−0.07, 0.23], BF_10_ = 0.02, OR = 1.08). Good profiles were identified with higher accuracy than Mixed profiles (Mdn = 1.34, HDI_89__%_ [−1.17, 1.51], BF_10_ > 999, OR = 3.83). The accuracy for the profiles is higher than chance level performance (33%), both overall and per condition (BF_10_s > 999).

#### Response Bias

A Bayesian categorical mixed-effects model was fitted, considering the effects of Condition (Reveal or Visible), and Profile (Good, Mixed, and Bad) on users’ profile type classification treating their answers as response bias. The responses to each profile were coded as follows: if the user selected “good” this was treated as + 1, if the user selected “mixed” this was treated as 0, and if the user selected “bad” this was treated as −1. A categorical cumulative model was employed as this treats the three responses as having an equal probability for each trial, unlike ordinal cumulative models which assume a latent continuous variable underlying each decision. See Table 3.

**TABLE 3:** Summary of the model fitted to the bias data, considering the experimental condition (Visible or Reveal) and Profile type (Good, Mixed, and Bad).

	β	89% HDI	PD	% in ROPE	*R̂*	BF_10_
Intercept [0]	1.57	[1.31, 1.82]	100.00	0.00	1.00	> 999
Intercept [1]	1.33	[1.00, 1.66]	100.00	0.00	1.00	> 999
[0] Condition [Visible]	0.06	[−0.29, 0.42]	60.31	57.18	1.00	0.05
[0] Profile [Good]	–0.83	[−1.24, −0.44]	99.99	0.20	1.00	43.64
[0] Profile [Mixed]	–2.89	[−3.16, −2.63]	100.00	0.00	1.00	> 999
[0] Condition [Visible]:Profile[Good]	–0.20	[−0.76, 0.37]	71.08	34.03	1.00	0.08
[0] Condition [Visible]:Profile[Mixed]	–0.02	[−0.39, 0.34]	54.08	57.16	1.00	0.05
[1] Condition [Visible]	–0.11	[−0.59, 0.34]	65.38	44.28	1.00	0.06
[1] Profile [Good]	–2.89	[−3.30, −2.49]	100.00	0.00	1.00	> 999
[1] Profile [Mixed]	–5.01	[−5.33, −4.69]	100.00	0.00	1.00	> 999
[1] Condition [Visible]:Profile [Good]	–0.03	[−0.59, 0.55]	53.33	39.24	1.00	0.07
[1] Condition [Visible]:Profile [Mixed]	0.30	[−0.14, 0.73]	86.24	29.69	1.00	0.10

**FIGURE 4:**
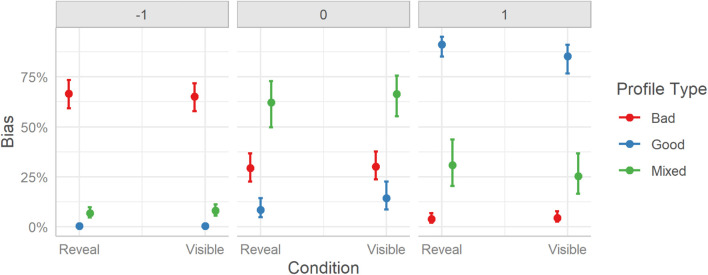
User response bias to rating the profiles between the two experimental conditions (Reveal and Visible) split over the three Profile conditions (Bad, Mixed, and Good). Error bars indicate 95% credible intervals.

The model posteriors for categorical models are difficult to interpret directly from the coefficients table. Plotting the marginal effects is a more appropriate method of unpacking the findings, see Figure 4.

Inspecting the marginal effects reveals a more detailed picture of users’ response patterns than if one considers only the overall difference. Bad profiles (67%) had the highest probability of being labeled by users as “bad” (−1), followed by Mixed profiles (7%) which had a much smaller but non-zero probability. Good profiles (0%) did not receive a “bad” label. The “mixed” (0) label was predominantly assigned to Mixed profiles (62%), with Bad profiles (29%) having the next highest probability, while Good profiles (8%) having a lower but non-zero probability. The “good” (+ 1) label was assigned with the highest probability to Good profiles (91%), followed by Mixed profiles (31%), and with low probability to Bad profiles (4%). All differences were significantly different, BF_10_s ≥ 45.

The response bias data echoes the finding that users are fairly accurate in their categorization but also suggests that when users were wrong in their classification, they tended to have an upward bias, as indicated by Mixed profiles being more likely to be misjudged as “good” instead of “bad.”

#### Judgment Confidence

To analyze the Likert-type data, a Bayesian ordinal mixed-effects model was fitted (using a cumulative probit likelihood), considering the effects of Condition (Reveal or Visible) and Profile (Good, Mixed, and Bad) on users’ judgment confidence ratings. The latent ordinal thresholds (i.e., nine intercepts) were estimated from a single fixed threshold (the lowest value) across users. The results are presented in Table 4.

**TABLE 4:** Summary of the model fitted to the judgment confidence data, considering the experimental condition (Visible or Reveal) and Profile type (Good, Mixed, and Bad).

	β	89% HDI	PD	% in ROPE	*R̂*	BF_10_
Intercept [1]	–3.92	[−4.20, −3.64]	100.00	0.00	1.00	> 999
Intercept [2]	–3.49	[−3.72, −3.26]	100.00	0.00	1.00	> 999
Intercept [3]	–2.85	[−3.06, −2.64]	100.00	0.00	1.00	> 999
Intercept [4]	–2.21	[−2.40, −2.00]	100.00	0.00	1.00	> 999
Intercept [5]	–1.55	[−1.74, −1.35]	100.00	0.00	1.00	> 999
Intercept [6]	–1.01	[−1.21, −0.82]	100.00	0.00	1.00	> 999
Intercept [7]	–0.29	[−0.49, −0.10]	99.24	5.31	1.00	0.46
Intercept [8]	0.56	[0.37, 0.75]	100.00	0.00	1.00	114.42
Intercept [9]	1.35	[1.16, 1.55]	100.00	0.00	1.00	> 999
Condition [Visible]	–0.18	[−0.46, 0.08]	86.16	26.15	1.00	0.06
Original [Good]	–0.29	[−0.34, −0.24]	100.00	0.00	1.00	> 999
Original [Mixed]	0.04	[−0.02, 0.09]	86.77	97.87	1.00	0.01
Condition [Visible]: Profile [Good]	–0.02	[−0.09, 0.05]	69.77	95.67	1.00	0.01
Condition [Visible]: Profile [Mixed]	–0.01	[−0.07, 0.07]	51.34	97.49	1.00	0.01

*Due to multicollinearity between thresholds, ROPE estimates may be unreliable.*

The coefficient *Condition [Visible]* indicates the extent to which people in the Visible condition differed from those in the Reveal condition (the reference category) on the latent scale of judgment confidence in their classification scores with reference to the Bad profile condition. Here, we find that the ratings users gave in both conditions were similar, as the 89% HDI crosses 0, the PD only indicated 86.16% directionality, the posterior distribution has 26.15% overlap with the ROPE, and the Bayes Factor indicates moderate evidence in favor of no difference (BF_01_ = 16.67). Additionally, the coefficients *Original [Good]* and *Original [Mixed]* indicate the extent to which users seeing Good and Mixed profiles differed in their confidence judgment ratings compared to seeing Bad profiles. Both indicate differences in judgment confidence. No interaction between Condition and Profile type seems to be present for judgment confidence. As such, the results focus on contrasts between the profile conditions.

Considering the difference in judgment confidence as a single relation effect (i.e., average difference in scores) we find that users were overall more confident in their judgments when seeing a Good profile than a Bad profile, Mdn = −0.16, HDI_89__%_ [−0.23, −0.09], BF_10_ = 3.05; however, the BF suggests only anecdotal-to-moderate evidence for a difference. Mixed profiles were judged with lower confidence than both Bad, Mdn = 0.25, HDI_89__%_ [0.17, 0.32], BF_10_ > 999, and Good profiles, Mdn = 0.84, HDI_89__%_ [0.33, −0.47], BF_10_ > 999.

**FIGURE 5:**
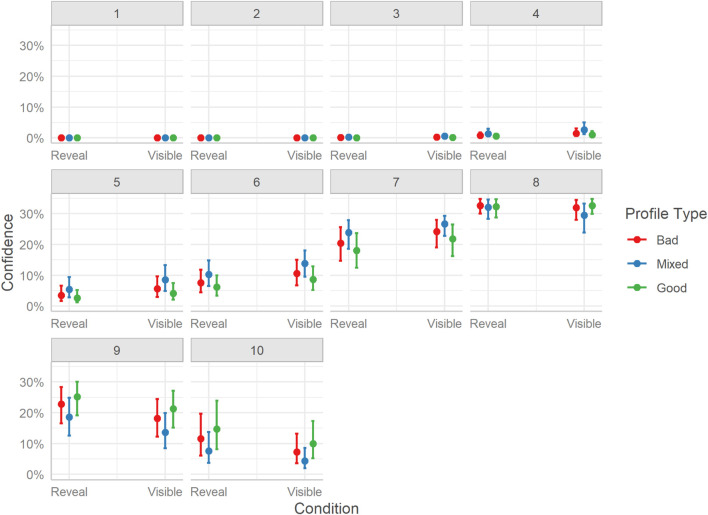
Marginal effects of judgment confidence for profile classification. The posterior mean estimates of the probability of responses in each of the three Profiles (Bad, Good, Mixed) by the two experimental Conditions (Reveal or Visible). Error bars indicate 95% credible intervals.

We also consider the distribution of responses because a single relation effect can obscure more subtle differences from polarizing responses (e.g., bimodality or variance differences). For brevity, we focus on the marginal effects displayed in Figure 5 to consider the distribution of responses across the scale, as reporting all pairwise contrast at each scale threshold would hinder interpretation and clarity. Here, we see that Good profiles resulted in more positive judgment ratings (higher probability of receiving scores of 8, 9, and 10). Mixed profiles seem to produce a more moderate response pattern with higher probabilities for ratings in the middle of the scale (i.e., 4-7). Bad profiles were rated with similar confidence to Good profiles, with ratings being more probable for the higher end of the scale (i.e., 7-10) although not quite as strongly, as more medium-scale values also being present (e.g., 6). The model also finds that the lower end of the scale (values between 1-4) was rarely used, suggesting that users were overall moderately to highly confident in all their judgments with only small differences between profile types.

#### Perceptions of Hosts

We now consider how the differences in profiles and experimental condition affected users’ perceptions of hosts on ratings of sociability, trustworthiness, and credibility. To analyze the Likert-type responses for each measure, we fit Bayesian ordinal mixed-effects models (using a cumulative probit likelihood), considering the effects of Condition (Reveal or Visible) and Profile (Good, Mixed, and Bad).

##### Sociability

The effects of condition and profile type on perceived sociability of the host are reported in Table 5.

**TABLE 5:** Summary of the model fitted to the sociability ratings, considering the experimental condition (Visible or Reveal) and Profile type (Good, Mixed, and Bad).

	β	89% HDI	PD	% in ROPE	*R̂*	BF_10_
Intercept [1]	–2.70	[−2.84, −2.55]	100.00	0.00	1.00	> 999
Intercept [2]	–2.15	[−2.29, −2.02]	100.00	0.00	1.00	> 999
Intercept [3]	–1.62	[−1.75, −1.49]	100.00	0.00	1.00	> 999
Intercept [4]	–1.11	[−1.24, −0.99]	100.00	0.00	1.00	> 999
Intercept [5]	–0.45	[−0.57, −0.32]	100.00	0.01	1.00	> 999
Intercept [6]	0.17	[0.05, 0.30]	98.73	16.52	1.00	0.38
Intercept [7]	0.81	[0.68, 0.93]	100.00	0.00	1.00	> 999
Intercept [8]	1.56	[1.43, 1.69]	100.00	0.00	1.00	> 999
Intercept [9]	2.44	[2.29, 2.58]	100.00	0.00	1.00	> 999
Condition [Visible]	–0.16	[−0.34, 0.01]	93.52	26.84	1.00	0.07
Original [Good]	–0.53	[−0.58, −0.48]	100.00	0.00	1.00	> 999
Original [Mixed]	–0.98	[−1.03, −0.93]	100.00	0.00	1.00	> 999
Condition [Visible]: Profile [Good]	–0.08	[−0.15, −0.01]	96.59	69.55	1.00	0.05
Condition [Visible]: Profile [Mixed]	–0.05	[−0.12, 0.02]	87.33	88.46	1.00	0.02

*Due to multicollinearity between thresholds, ROPE estimates may be unreliable.*

Considering the difference in sociability ratings as a single relation effect we find that users rated hosts of Bad profiles lower than both hosts of Mixed, Mdn = −1.57, HDI_89__%_ [−1.65, −1.5], BF_10_ > 999, and Good profiles, Mdn = −0.82, HDI_89__%_ [−0.89, −0.75], BF_10_ > 1000. Unexpectedly, users rated Mixed profile hosts as more sociable than Good profile hosts, Mdn = 0.75, HDI_89__%_ [0.68, 0.82], BF_10_ > 999.

**FIGURE 6:**
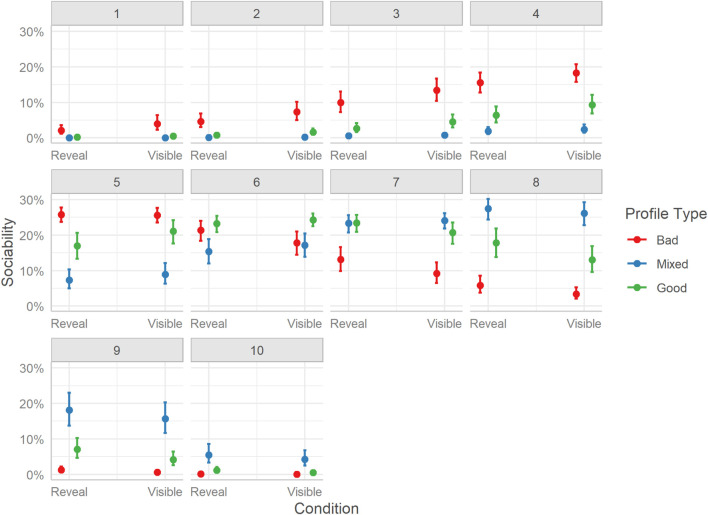
Marginal effects of sociability. The posterior mean estimates of the probability of responses in each of the three Profiles (Bad, Good, Mixed) by the two experimental Conditions (Reveal or Visible). Error bars indicate 95% credible intervals.

Looking at the distributions of ratings, the effects are more complex (Figure 6). It is clear that Bad profiles had a higher probability of being rated as having less sociable hosts (ratings between 1 and 5); however, Good profiles while being less probable to receive ratings on the lower end of the scale, show no clear pattern for the rating probability, with moderate scores (e.g., 5) being as likely as higher scores (e.g., 8). Mixed profile hosts, surprisingly, while also having a wider distribution of scores had higher probabilities for the top end of the scale (i.e., 8-10), which resulted in the overall higher rating.

##### Trustworthiness

The effects of condition and profile type on perceived trustworthiness of the host are reported in Table 6.

**TABLE 6:** Summary of the model fitted to the trustworthiness ratings, considering the experimental condition (Visible or Reveal) and Profile type (Good, Mixed, and Bad).

	β	89% HDI	PD	% in ROPE	*R̂*	BF_10_
Intercept [1]	–2.76	[−2.91, −2.60]	100.00	0.00	1.00	> 999
Intercept [2]	–2.10	[−2.24, −1.97]	100.00	0.00	1.00	> 999
Intercept [3]	–1.59	[−1.72, −1.46]	100.00	0.00	1.00	> 999
Intercept [4]	–1.15	[−1.27, −1.01]	100.00	0.00	1.00	> 999
Intercept [5]	–0.51	[−0.64, −0.38]	100.00	0.01	1.00	> 999
Intercept [6]	0.06	[−0.07, 0.19]	76.65	68.10	1.00	0.04
Intercept [7]	0.68	[0.55, 0.81]	100.00	0.00	1.00	> 999
Intercept [8]	1.46	[1.33, 1.60]	100.00	0.00	1.00	> 999
Intercept [9]	2.35	[2.21, 2.50]	100.00	0.00	1.00	> 999
Condition [Visible]	–0.21	[−0.39, −0.03]	96.95	16.09	1.00	0.13
Original [Good]	–0.58	[−0.63, −0.53]	100.00	0.00	1.00	> 999
Original [Mixed]	–1.03	[−1.08, −0.98]	100.00	0.00	1.00	> 999
Condition [Visible]: Profile [Good]	–0.01	[−0.06, 0.08]	58.10	97.80	1.00	0.01
Condition [Visible]: Profile [Mixed]	0.03	[−0.04, 0.09]	73.28	95.61	1.00	0.01

*Due to multicollinearity between thresholds, ROPE estimates may be unreliable.*

The differences in trustworthiness ratings mirror those of sociability. Overall, users rated hosts of Bad profiles lower than both hosts of Mixed profiles, Mdn = −1.67, HDI_89__%_ [−1.75, −1.60], BF_10_ > 999, and hosts of Good profiles, Mdn = −0.85, HDI_89__%_ [−0.92, −0.78], BF_10_ > 1000. Similarly, users rated Mixed profile hosts as more trustworthy than Good profile hosts, Mdn = 0.82, HDI_89__%_ [0.75, 0.89], BF_10_ > 1000.

**FIGURE 7:**
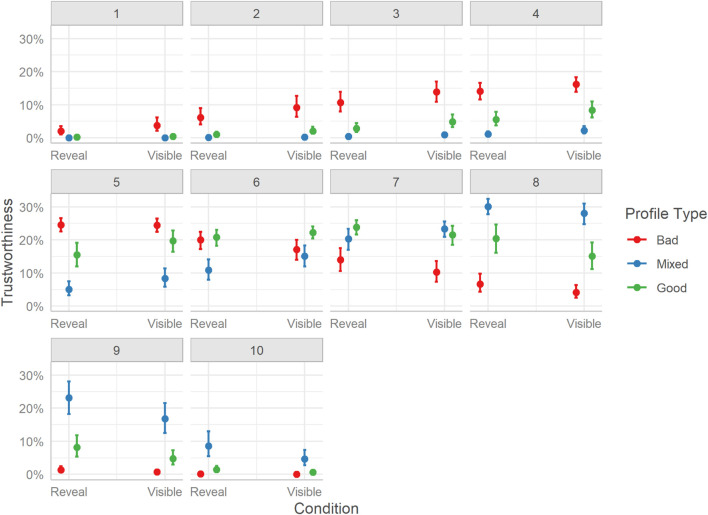
Marginal effects of trustworthiness. The posterior mean estimates of the probability of responses in each of the three Profiles (Bad, Good, Mixed) by the two experimental Conditions (Reveal or Visible). Error bars indicate 95% credible intervals.

The distribution of trustworthiness ratings is less clear than for sociability, yet overall follows a similar pattern. Although Bad profiles had a higher probability of receiving lower-end ratings (e.g., 1-4), they also had a high probability of receiving more moderate ratings, especially a rating of 5, which may indicate more uncertainty for this dimension. Yet, the probability for higher-end ratings was very small. For Mixed profiles, lower-end ratings were less probable as were more moderate ratings (e.g., 5,6), yet the probabilities of higher values were progressively higher, plateauing around 8. For Good profiles, the more moderate ratings were more probable (i.e., 5-8), with lower-end or higher-end ratings being less probable. See Figure 7.

##### Credibility

The effects of condition and profile type on perceived credibility of the information provided by the host are presented in Table 7.

**TABLE 7:** Summary of the model fitted to the credibility ratings, considering the experimental condition (Visible or Reveal) and Profile type (Good, Mixed, and Bad).

	β	89% HDI	PD	% in ROPE	*R̂*	BF_10_
Intercept [1]	–3.37	[−3.60, −3.14]	100.00	0.00	1.00	> 999
Intercept [2]	–2.89	[−3.09, −2.67]	100.00	0.00	1.00	> 999
Intercept [3]	–2.48	[−2.68, −2.28]	100.00	0.00	1.00	> 999
Intercept [4]	–2.11	[−2.31, −1.91]	100.00	0.00	1.00	> 999
Intercept [5]	–1.38	[−1.57, −1.18]	100.00	0.01	1.00	> 999
Intercept [6]	–0.77	[−0.96, −0.58]	100.00	0.01	1.00	> 999
Intercept [7]	0.09	[−0.11, 0.28]	76.60	48.11	1.00	0.03
Intercept [8]	1.00	[0.81, 1.19]	100.00	0.00	1.00	> 999
Intercept [9]	2.05	[1.84, 2.24]	100.00	0.00	1.00	> 999
Condition [Visible]	–0.31	[−0.59, −0.03]	96.02	10.70	1.00	0.16
Original [Good]	–0.51	[−0.56, −0.46]	100.00	0.00	1.00	> 999
Original [Mixed]	–0.60	[−0.65, −0.54]	100.00	0.00	1.00	> 999
Condition [Visible]: Profile [Good]	–0.03	[−0.10, 0.04]	74.03	94.61	1.00	0.01
Condition [Visible]: Profile [Mixed]	0.03	[−0.04, 0.10]	75.08	94.40	1.00	0.01

*Due to multicollinearity between thresholds, ROPE estimates may be unreliable.*

Considering overall differences in credibility ratings of hosts, we see the same pattern as with the other two host perception metrics. Bad profile hosts received lower ratings than Mixed profile hosts, Mdn = −1.09, HDI_89__%_ [−1.16, −1.02], BF_10_ > 999, and Good profile hosts, Mdn = −0.37, HDI_89__%_ [−0.44, −0.30], BF_10_ > 999. Also, users rated Mixed profile hosts as more credible in the information, they provided than Good profile hosts, Mdn = 0.72, HDI_89__%_ [0.65, 0.80], BF_10_ > 1000.

**FIGURE 8:**
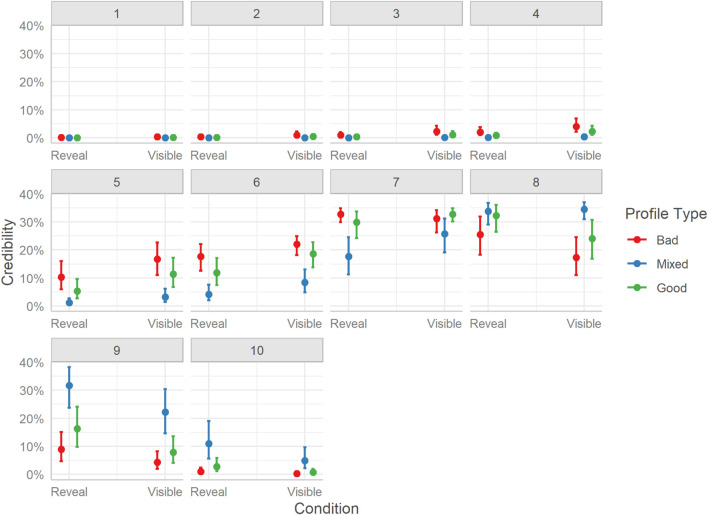
Marginal effects of credibility. The posterior mean estimates of the probability of responses in each of the three Profiles (Bad, Good, Mixed) by the two experimental Conditions (Reveal or Visible). Error bars indicate 95% credible intervals.

The distribution of credibility ratings follows a more compact pattern, with most of the differences in probabilities occurring toward the end of the scale. Bad profiles were more likely to receive lower-end (2-4) or moderate (5-7) ratings. Mixed profiles had the highest probability of obtaining high-end ratings (8-10), and very low or zero probability of receiving lower-end ratings. Good profiles showed more variability in ratings, being more clustered around the middle of the credibility scale (5-8), with very few ratings at either end of the scale. Surprisingly, even for Bad profiles, which contained poor reviews and comments, the lower end of the scale was rarely used. See Figure 8.

##### Relationship Between Measures

Considering the overall similarity in ratings across the three host perception metrics, it is important to consider how strongly users’ judgments correlate. High correlations overall may indicate that users’ impressions tended to be uniform (i.e., once they decided the quality of a room this produced an overall effect on their subsequent ratings), while more moderate correlations may suggest that different information was influential in their per room judgments.

Kendall Tau (tau-b; range: −1 to 1; 0.10-0.30 small, 0.30-0.50 medium, ≥ 0.50 large) Bayesian non-parametric correlations were conducted on the three host perception measures (Table 8). This was done as the data were originally bounded (here, ordered), and univariate and bivariate normality tests indicated deviations from normality in several correlations. Kendall correlations are typically more robust and efficient than alternative non-parametric approaches (e.g., Spearman’s Rank correlation), and are also available under a Bayesian framework.

**TABLE 8:** Correlations Between Perception of Hosts Measures for Each Profile Condition.

Profile		Kendall’s Tau	BF_10_	*p*-value	89% HDI	
***Bad***						

Sociability	-Trustworthiness	0.72***, + + +	> 999	< 0.001	[0.60	0.79]
Sociability	- Credibility	0.23***, + +	96.62	< 0.001	[0.12	0.32]
Credibility	-Trustworthiness	0.32***, + + +	> 999	< 0.001	[0.22	0.41]

***Mixed***						

Sociability	- Trustworthiness	0.71***, + + +	> 999	< 0.001	[0.59	0.78]
Sociability	- Credibility	0.40***, + + +	> 999	< 0.001	[0.29	0.48]
Credibility	-Trustworthiness	0.50***, + + +	> 999	< 0.001	[0.39	0.58]

***Good***						

Sociability	- Trustworthiness	0.78***, + + +	> 999	< 0.001	[0.65	0.84]
Sociability	- Credibility	0.61***, + + +	> 999	< 0.001	[0.49	0.69]
Credibility	-Trustworthiness	0.70***, + + +	> 999	< 0.001	[0.58	0.77]

* p < 0.05, ** p < 0.01, ***p < 0.001; + BF10 > 10, ++ BF10 > 30, +++ BF10 > 100.

There were medium-to-large positive correlations across all measures and profile types. Of note, the strength of the correlations is weaker for the Bad and Mixed profiles, which would indicate that when information about room quality was less positive, decisions were more varied on the host perception metrics. Conversely, when seeing a Good profile, an overall halo-type effect seems to occur where ratings on all metrics have a strong positive relationship.

Although all correlations are significant and positive, we explored whether the strength of the correlations between the three measures differed in magnitude. Steiger’s Z tests for differences between correlations were conducted (see Supplementary Materials for the full results). This revealed that the sociability-trustworthiness (S-T) correlations were consistent across all three profile conditions, but there were differences for credibility-sociability (C-S) between Bad-Good and Mixed-Good, as well as for credibility-trustworthiness (C-T) between Bad-Good and Mixed-Good. Furthermore, it was revealed that the S-T correlation was stronger than both the C-T and C-S correlations, in both Bad and Mixed profiles. While for Good profiles the S-T correlation was significantly stronger than the C-S, but not C-T, correlation. Speculatively, this suggests that users view information credibility as a separate dimension from both trustworthiness and sociability, which reflects their different nature, as the latter two are character traits of the host, while the former encompasses the entirety of the information on each profile (i.e., host and other users).

### Discussion

In contrast to our predictions, data provide no support for a difference in user responses as a product of the experimental condition – Visible or Reveal – suggesting that seeing seven TRI elements or three does not impact decision-making or host perceptions (**H_1_**). The lack of differences was observed in all measured variables (**H_2_, H_3_**), supporting an information discounting explanation (as found in Zloteanu et al., 2018; see also Harries and Harries, 2001). Namely, it would seem that the judgments users make are unaffected by the quantity of TRI elements they have available.

When considering differences per the quality of the profiles (**H_4_**), users displayed a fairly high ability to distinguish profile quality (70% overall), suggesting that the diagnostic information contained in the various TRIs was relevant to their SE decision-making. However, users seem to display an upward bias in their perceptions of profile quality. Specifically, when they misidentified the quality of a profile the mistake tended to favor a more positive perception. Users were also the least confident when rating Mixed profiles, while showing high confidence for Good and Bad profiles. This may reflect the more challenging identification task, as mixed profiles could be interpreted as either bad or good, while the latter would most likely only be confused as mixed.

Regarding differences in host perceptions among the three profile conditions, the results present both a confirmation of our predictions and novel insights. While ratings between the Good and Bad profile conditions were in the expected direction for all metrics, the Mixed profile condition received higher ratings on all three metrics, indicating that the TRI content of such profiles made users have more positive perceptions of hosts, even higher than when judging good profiles.

The results also show an interesting pattern for the mixed quality profiles, as users displayed the lowest accuracy and judgment confidence, but gave the highest ratings on the three host perception metrics. Mixed profiles may have been more challenging, as they could be (mis)interpreted as both of higher or lower quality, yet they provide a contrast to good profiles which may be seen as more hotel-like and impersonal, conflicting with the SE mentality of “feel at home” (Tussyadiah and Zach, 2017). Thus, while users were more eager to rent good profiles than mixed, they had more positive impressions of mixed profiles which may have met their expectations. We speculate that the increased variability and/or uncertainty of the mixed profiles may have made them seem more “believable” or “realistic,” leading to sympathy toward the host and the inflation in ratings. This resonates with research on users preferring more authentic-style accommodations for their SE experiences (Mody et al., 2019). Should our speculation be confirmed, it would provide a rather stark contrast with the actual outcomes seen in SE platforms, whose “5-for-5” ethos – somewhat encouraged by the platforms’ design – ultimately leads almost all profiles to appear “above average” (Zaki et al., 2021; Zervas et al., 2021). Future research may consider investigating such an effect, especially in light of the overly positive information typically featured on SE platforms.

## Study 2: Research Questions and Hypotheses

Given the results of Study 1, we were interested in uncovering whether the TRI elements selected or avoided by users have any bearing on their final judgments and host perceptions. Namely, do certain combinations of elements confer more diagnosticity and aid accurate decision-making? For this we analyzed the selection pattern of Study 1’s Reveal condition and extracted the six most selected TRI pairs and the six most avoided pairs (see SM); pairs were selected instead of triplets as the results of Study 1 indicated parity in accuracy scores even with three TRIs, leaving little variability. Our hypotheses were as follows:

**H_5_**: Users will be more accurate in detecting profile quality when presented with TRI that is typically desired (Wanted TRI) than undesired (Avoided TRI).
**H_6_**: Users in the Wanted TRI condition will be more polarized in their rental of bad (i.e., fewer) and good (i.e., more) rooms.
**H_7_**: The Wanted TRI group will be more confident in their judgments.
**H_8_**: Users in the Wanted TRI group will be more positively biased than users in the Avoided TRI group.
**H_9_**: The Wanted TRI group will have more positive host perceptions overall than users in the Avoided TRI group.
**H_10_**: As in Study 1, we assume there will be differences in the judgment of the three profile types.

### Method

For the same reasons as in Study 1, we used an experimental approach to address the hypotheses in this study.

#### Participants and Design

A mixed design was employed, with Condition (Wanted or Avoided) as the between-participants factor, and Profile (Good, Mixed, and Bad) as the within-participants factor. The same seven dependent measures as in Study 1 were used: profile quality, rent decision, judgment confidence, sociability, credibility, trustworthiness, accuracy, and bias.

A total of 354 participants were collected (one participant’s demographics were not recorded, although their experimental data were; 191 males, 162 females; *M_*Age*_* = 29.31, *SD* = 10.15; Avoided condition *n* = 174, Wanted condition *n* = 180) through mTurk in exchange for a flat fee of $1.00. A sensitivity analysis for a 2 × 3 factorial interaction set at 80% power and alpha of 0.05 (two-sided), estimates that effect sizes as small as Cohen’s *f* = 0.12 or $\eta_{P}^{2}$ = 0.01 can be reliably detected with the current sample size. Informed consent was obtained from all participants.

#### Materials and Stimuli

The stimuli used mirrored those of Study 1 with the modification that in both experimental groups – Avoided and Wanted – the profiles displayed two TRI elements per profile. In the Avoided condition, the six combinations were: social media + star ratings, social media + number of reviews, social media + reviews guests, social media + reviews hosts, reviews hosts + number of reviews, reviews hosts + cross-platform reputation. In the Wanted condition, the six combinations were: star ratings + number of reviews, star ratings + reviews guests, reviews guests + number of reviews, star ratings + reviews hosts, reviews guests + cross-platform reputation, reviews guests + reviews hosts. Each condition-specific profile was generated ad lib and contained one random TRI duo from the respective lists. There were 30 profiles per group (10 × Good, 10 × Mixed, 10 × Bad).

#### Procedure

The same procedure as in Study 1’s Visible condition was followed, with the difference that users were randomly allocated to either the Avoided or Wanted group (the pseudo-randomizer ensured that the TRI duos would be presented in equal numbers across the sample). At the start, users saw the same example profile (explaining what each potential TRI element they would see contained) and engaged in a six-profile practice trial to ensure they understood the classification task. The remainder of the task was identical to Study 1, as were the dependent measures.

#### Data Analysis

The same modeling protocols as in Study 1 were employed for each of the reported dependent variables. Considering that Study 2 contained multiple TRI duos in both the Avoided and Wanted experimental groups, more maximal models including the duo subtypes as a random factor were considered, however these posed model convergence issues, and if compared to the final models did not provide any explanatory benefits, introducing only more complexity and error. As such the final models have the same structure as those in Study 1, with fixed effects for Condition and Profile, and random effects for participants.

### Results

User responses were analyzed based on the two experimental groups (Condition: Wanted and Avoided) and the three profile conditions (Profile: Good, Mixed, and Bad).

#### Decision to Rent

The model considering the effects of Condition and Profile on rent decisions is reported in Table 9.

**TABLE 9:** Summary of the model fitted to the rent data, considering the experimental condition (Wanted or Avoided) and Profile type (Good, Mixed, and Bad).

	β	89% HDI	PD	% in ROPE	*R̂*	BF_10_
Intercept	–0.24	[−0.31, −0.15]	100.00	13.66	1.00	>999
Condition [Wanted]	0.02	[−0.08, 0.12]	63.26	99.44	1.00	0.66
Profile [Good]	–1.06	[−1.12, −1.00]	100.00	0.00	1.00	>999
Profile [Mixed]	–1.81	[−1.88, −1.74]	100.00	0.00	1.00	>999
Condition [Wanted]: Profile [Good]	–0.14	[−0.22, −0.06]	99.74	81.36	1.00	25.12
Condition [Wanted]: Profile [Mixed]	–0.28	[−0.36, −0.19]	98.88	3.80	1.00	>999

*Results are given on the logit (not the response) scale.*

**FIGURE 9:**
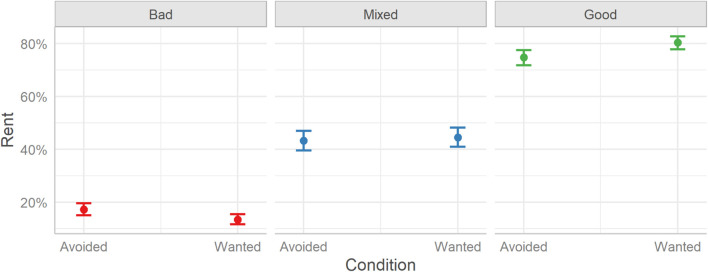
The probabilities of deciding to rent a room between the two experimental conditions (Wanted and Avoided) split over the three Profile conditions (Bad, Mixed, and Good). Error bars indicate 95% credible intervals.

The model shows differences in rent decisions between the three profile conditions but no overall effect of Condition. The model also indicates a potential interaction effect between Condition and Profile, however, although the BFs show support for an interaction it should be noted that the effect overlaps with the ROPE which suggests it is potentially no different from 0. To unpack the above effects, we conduct simple effects and pairwise contrasts and plot the marginal effects for the rent decisions. See Figure 9.

First, we consider the difference between the three profile conditions, unpacked between the two experimental groups. In the Avoided group, users decided to rent fewer Bad rooms (17%) than both Good (75%), Mdn = −2.66, HDI_89__%_ [−2.79, −2.53], BF_10_ > 999, OR = 0.07, and Mixed (43%), Mdn = −1.30, HDI_89__%_ [−1.42, −1.18], BF_10_ > 999, OR = 0.27. They also rented more Good than Mixed rooms, Mdn = 1.36, HDI_89__%_ [1.24, 1.47], BF_10_ > 999, OR = 3.89). In the Wanted group, users showed an identical pattern of results (Bad (13%) – Good (80%), Mdn = −3.28, HDI_89__%_ [−3.41, −3.14], BF_10_ > 999, OR = 0.04; Bad – Mixed (45%), Mdn = −1.64, HDI_89__%_ [−1.76, −1.52], BF_10_ > 999, OR = 0.19; Good-Mixed, Mdn = 1.63, HDI_89__%_ [1.52, 1.75], BF_10_ > 999, OR = 5.13).

Second, we consider the difference between the two experimental groups, unpacked between the three profile conditions. A difference in decisions to rent Bad profiles was observed between the Avoided (17%) and Wanted (13%) groups, with the latter renting fewer such rooms, Mdn = 0.29, HDI_89__%_ [0.12, 0.47], BF_10_ = 19.27, OR = 1.34. Similarly, a difference was observed for renting Good profiles, with users in the Avoided group (75%) renting fewer than users in Wanted group (80%), Mdn = −0.33, HDI_89__%_ [−0.50, −0.16], BF_10_ = 69.93, OR = 0.72. However, no reliable difference between groups was found when deciding to rent Mixed rooms, Mdn = −0.05, HDI_89__%_ [−0.22, 0.11], BF_10_ = 0.64, OR = 0.95.

#### Accuracy

The model considering the effects of Condition and Profile on user accuracy is reported in Table 10.

**TABLE 10:** Summary of the model fitted to the accuracy data, considering the experimental condition (Wanted or Avoided) and Profile type (Good, Mixed, and Bad).

	β	89% HDI	PD	% in ROPE	*R̂*	BF_10_
Intercept	0.73	[0.66, 0.80]	100.00	0.00	1.00	> 999
Condition [Wanted]	0.25	[0.15, 0.35]	100.00	12.12	1.00	45.50
Profile [Good]	–0.79	[−0.86, −0.73]	100.00	0.00	1.00	> 999
Profile [Mixed]	–0.33	[−0.39, −0.27]	100.00	0.00	1.00	> 999
Condition [Wanted]: Profile [Good]	–0.18	[−0.26, −0.08]	99.90	54.36	1.00	1.50
Condition [Wanted]: Profile [Mixed]	–0.12	[−0.21, −0.04]	98.95	86.05	1.00	0.16

*Results are given on the logit (not the response) scale.*

**FIGURE 10:**
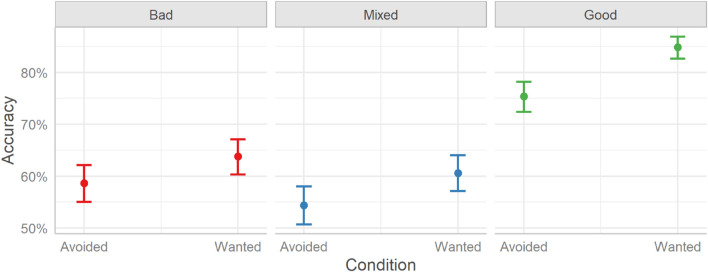
User accuracy in correctly categorizing a profile as bad, mixed, or good between the two experimental conditions (Avoided and Wanted) split over the three Profile conditions (Bad, Mixed, and Good). Error bars indicate 95% credible intervals.

The model shows that accuracy was affected by the condition in which users were placed, Avoided or Wanted, and based on the type of profile they saw, Good, Mixed, or Bad. Although the model suggests an interaction may be present (see HDIs and PDs), the indices for significance do not show strong evidence for an effect (see ROPEs and BFs). As such, we focus on the overall effect of Condition and the contrasts between the Profile types. See Figure 10.

The marginal effects revealed that there is indeed a difference in accuracy based on Condition, with users seeing the Avoided TRI elements showing overall lower accuracy (63%) than users seeing the Wanted TRI elements (70%) across all profile types, Mdn = −0.36, HDI_89__%_ [−0.50, −0.22], BF_10_ = 34.29, OR = 0.70. Considering the difference in accuracy between profiles, accuracy was lower for Bad profiles (62%) than Good profiles (80%), Mdn = −0.96, HDI_89__%_ [−1.06, −0.87], BF_10_ > 999, OR = 0.38, but higher than Mixed profiles (58%), Mdn = 0.15, HDI_89__%_ [0.07, 0.24], BF_10_ = 0.63, OR = 1.17; although, the evidence in favor of a difference is anecdotal. Good profiles were also rated with higher accuracy than Mixed profiles, Mdn = 1.12, HDI_89__%_ [1.03, 1.21], BF_10_ > 999, OR = 3.06.

#### Response Bias

The model considering the effects of Condition and Profile on users’ response bias is reported in Table 11. The same response coding as in Study 1 was used.

**TABLE 11:** Summary of the model fitted to the bias data, considering the experimental condition (Wanted or Avoided) and Profile type (Good, Mixed, and Bad).

	β	89% HDI	PD	% in ROPE	*R̂*	BF_10_
Intercept [0]	1.09	[0.99, 1.19]	100.00	0.00	1.00	> 999
Intercept [1]	0.67	[0.54, 0.80]	100.00	0.00	1.00	> 999
[0] Condition [Wanted]	0.19	[0.05, 0.33]	98.57	46.04	1.00	0.19
[0] Profile [Good]	–0.54	[−0.70, −0.39]	100.00	0.00	1.00	> 999
[0] Profile [Mixed]	–2.11	[−2.22, −2.00]	100.00	0.00	1.00	> 999
[0] Condition[Wanted]:Profile[Good]	0.02	[−0.20, 0.25]	56.17	80.45	1.00	0.03
[0] Condition[Wanted]:Profile[Mixed]	–0.35	[−0.50, −0.20]	99.99	3.16	1.00	24.76
[1] Condition [Visible]	0.17	[−0.02, 0.35]	92.68	54.70	1.00	0.07
[1] Profile [Good]	–2.30	[−2.46, −2.14]	100.00	0.00	1.00	> 999
[1] Profile [Mixed]	–4.14	[−4.28, −3.99]	100.00	0.00	1.00	> 999
[1] Condition[Wanted]:Profile[Good]	–0.36	[−0.58, −0.14]	99.65	9.01	1.00	1.03
[1] Condition[Wanted]:Profile[Mixed]	–0.81	[−1.01, −0.62]	100.00	0.00	1.00	> 999

**FIGURE 11:**
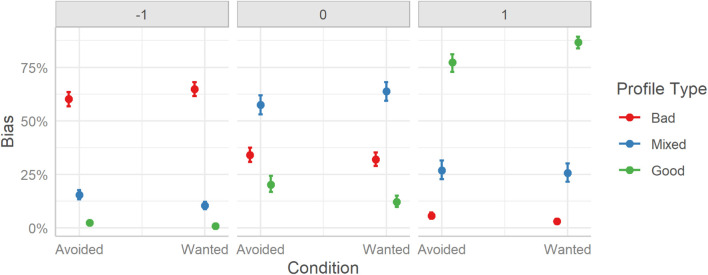
User response bias to rating the profiles between the two experimental conditions (Avoided and Wanted) split over the three Profile conditions (Bad, Mixed, and Good). Error bars indicate 95% credible intervals.

As mentioned previously, to permit interpretation of the results the marginal effects at each level of Profile and Condition were plotted. See Figure 11.

Considering differences between the three Profile types, the marginal effects reveal a similar pattern for response differences as in Study 1. Users, across both TRI conditions, were more likely to rate Bad profiles as “bad” (63%), Mixed profiles as “mixed” (61%), and Good profiles as “good” (82%). Similarly, as in Study 1, an upward bias in misclassifications was observed across both experimental groups [e.g., more likely to misclassify a Mixed profile as “good” (27%) instead of “bad” (13%)]. The difference between profile classification rates was significantly and strongly different within each response category, BFs > 999.

Considering differences between TRI groups, in line with our predictions, the Wanted group (72%) had better classification rates overall compared to the Avoided group (65%). By extension, when misclassifying a profile, users in the Wanted group tended to make fewer mistakes than users in the Avoided group. However, the pattern is less well-defined when considering Profile by Condition, with some showing little evidence of a difference (e.g., responding to Bad profiles with a “bad” label, Avoided (60%) vs. Wanted (65%), BF_10_ = 0.19), while other showing strong evidence (e.g., labeling a Good profile as “good” was higher for users that saw Wanted TRI elements (77%) than Avoided TRI element (87%), BF_10_ = 47.15, as was for labeling Mixed profiles as “mixed,” Avoided (58%) vs. Wanted (64%), BF_10_ = 16.12).

#### Judgment Confidence

The model considering the effects of Condition and Profile on judgment confidence is reported in Table 12.

**TABLE 12:** Summary of the model fitted to the judgment confidence data, considering the experimental conditions (Avoided or Wanted) and Profile type (Good, Mixed, and Bad).

	β	89% HDI	PD	%inROPE	*R̂*	BF_10_
Intercept [1]	–3.33	[−3.45, −3.21]	100.00	0.00	1.00	> 999
Intercept [2]	–2.93	[−3.04, −2.83]	100.00	0.00	1.00	> 999
Intercept [3]	–2.57	[−2.67, −2.47]	100.00	0.00	1.00	> 999
Intercept [4]	–2.11	[−2.21, −2.02]	100.00	0.00	1.00	> 999
Intercept [5]	–1.60	[−1.69, −1.51]	100.00	0.00	1.00	> 999
Intercept [6]	–0.98	[−1.07, −0.89]	100.00	0.00	1.00	> 999
Intercept [7]	–0.20	[−0.29, −0.11]	99.99	3.08	1.00	5.81
Intercept [8]	0.66	[0.57, 0.75]	100.00	0.00	1.00	> 999
Intercept [9]	1.39	[1.29, 1.48]	100.00	0.00	1.00	> 999
Condition [Wanted]	0.01	[−0.11, 0.13]	55.91	80.17	1.00	0.02
Original [Good]	–0.25	[−0.28, −0.22]	100.00	0.00	1.00	> 999
Original [Mixed]	0.10	[0.07, 0.12]	100.00	59.55	1.00	> 999
Condition [Wanted]: Profile [Good]	–0.09	[−0.13, −0.05]	99.99	65.71	1.00	4.07
Condition [Wanted]: Profile [Mixed]	–0.02	[−0.06, 0.02]	79.95	99.90	1.00	0.01

*Due to multicollinearity between thresholds, ROPE estimates may be unreliable.*

**FIGURE 12:**
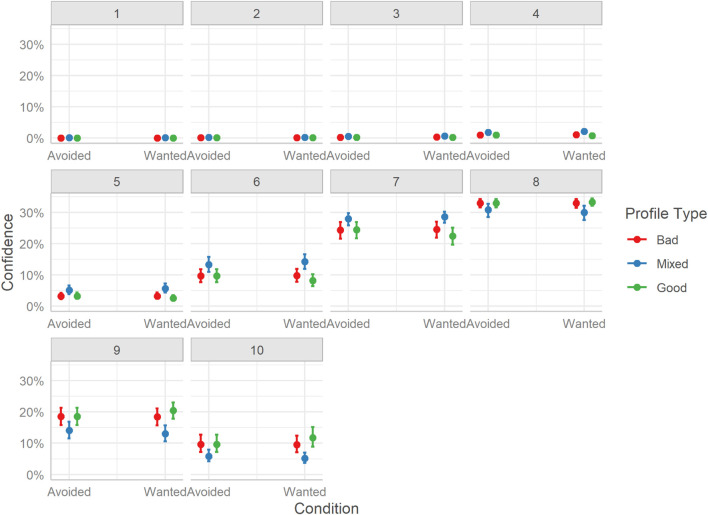
Marginal effects of judgment confidence for profile classification. The posterior mean estimates of the probability of responses in each of the three Profiles (Bad, Good, Mixed) by the two experimental Conditions (Avoided or Wanted). Error bars indicate 95% credible intervals.

The model indicates little support for a difference in judgment confidence between the two TRI groups and equivocal evidence for an interaction effect with profile type. Indeed, considering the difference between the two TRI groups at every Profile level did not reveal any differences (BF_10_s ≤ 0.02). As such, we focus on the difference in confidence between the three profile types (Figure 12).

Mirroring the findings of Study 1, users were more confident overall when judging Good profiles than Bad profiles, however, quantifying the evidence shows stronger support for no difference, Mdn = −0.06, HDI_89__%_ [−0.10, −0.02], BF_10_ = 0.07. And overall, users were less confident judging Mixed profiles than both Good, Mdn = 0.36, HDI_89__%_ [0.32, 0.40], BF_10_ > 999, and Bad profiles, Mdn = 0.30, HDI_89__%_ [0.26, 0.34], BF_10_ > 999.

Considering the distribution of ratings, the Good profiles tended to receive more ratings on the upper end of the scale (i.e., 8-10), as did Bad profiles (i.e., 8-10), while Mixed profiles received more moderate ratings (i.e., 5-7). As in Study 1, users seem to have been overall confident in their judgments, as the lower end of the scale (i.e., 1-4) was rarely employed.

#### Perceptions of Hosts

Given the difference in TRI elements between the Avoided and Wanted user groups, we consider how the information and perception of these elements may have impacted users’ ratings of hosts on perceived sociability, trustworthiness, and credibility.

##### Sociability

The model considering the effects of Condition and Profile on users’ ratings of perceived host sociability is reported in Table 13.

**TABLE 13:** Summary of the model fitted to the sociability ratings, considering the experimental condition (Avoided or Wanted) and Profile type (Good, Mixed, and Bad).

	β	89% HDI	PD	% in ROPE	*R̂*	BF_10_
Intercept [1]	–2.53	[−2.61, −2.46]	100.00	0.00	1.00	> 999
Intercept [2]	–2.03	[−2.10, −1.97]	100.00	0.00	1.00	> 999
Intercept [3]	–1.52	[−1.59, −1.46]	100.00	0.00	1.00	> 999
Intercept [4]	–1.04	[−1.10, −0.98]	100.00	0.00	1.00	> 999
Intercept [5]	–0.41	[−0.47, −0.35]	100.00	0.00	1.00	> 999
Intercept [6]	0.21	[0.15, 0.27]	100.00	0.20	1.00	> 999
Intercept [7]	0.92	[0.86, 0.99]	100.00	0.00	1.00	> 999
Intercept [8]	1.70	[1.63, 1.76]	100.00	0.00	1.00	> 999
Intercept [9]	2.40	[2.33, 2.48]	100.00	0.00	1.00	> 999
Condition [Wanted]	0.01	[−0.07, 0.09]	55.50	94.33	1.00	0.01
Original [Good]	–0.50	[−0.53, −0.47]	100.00	0.00	1.00	> 999
Original [Mixed]	–0.90	[−0.93, −0.88]	100.00	0.00	1.00	> 999
Condition [Wanted]: Profile [Good]	–0.04	[−0.08, 0.00]	95.25	99.22	1.00	0.02
Condition [Wanted]: Profile [Mixed]	–0.06	[−0.10, −0.02]	99.25	95.03	1.00	0.10

*Due to multicollinearity between thresholds, ROPE estimates may be unreliable.*

The model revealed no evidence of TRI condition influencing overall ratings of host sociability nor an interaction with profile type. As such, we focus on the differences between the three profile conditions. Mirroring the findings of Study 1, considering overall differences in ratings, users rated hosts of Bad profiles lower than those of Mixed profiles, Mdn = −0.75, HDI_89__%_ [−0.79, −0.71], BF_10_ > 999, and Good profiles, Mdn = −1.46, HDI_89__%_ [−1.51, −1.42], BF_10_ > 999. Unlike in Study 1, however, Good profiles were rated as having more sociable hosts compared to Mixed profiles, Mdn = 0.71, HDI_89__%_ [0.67, 0.75], BF_10_ > 999.

**FIGURE 13:**
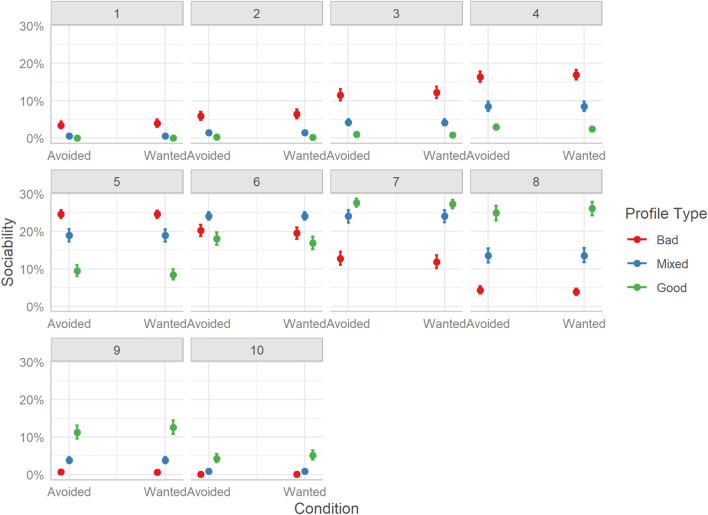
Marginal effects of sociability. The posterior mean estimates of the probability of responses in each of the three Profiles (Bad, Good, Mixed) by the two experimental Conditions (Avoided or Wanted). Error bars indicate 95% credible intervals.

Considering the distribution of sociability ratings, we see clearly that Bad profiles were more likely to receive lower end ratings (i.e., 1-4) and moderate ratings (i.e., 5-6). Mixed profiles were more likely to be rated with values between 5 and 8. Also, Good profiles received almost no lower-end ratings and predominantly higher-end values (i.e., 8-10). See Figure 13.

##### Trustworthiness

The model considering the effects of Condition and Profile on users’ ratings of perceived host trustworthiness is reported in Table 14.

**TABLE 14:** Summary of the model fitted to the trustworthiness ratings, considering the experimental condition (Avoided or Wanted) and Profile type (Good, Mixed, and Bad).

	β	89% HDI	PD	% in ROPE	*R̂*	BF_10_
Intercept [1]	–2.63	[−2.71, −2.56]	100.00	0.00	1.00	> 999
Intercept [2]	–2.12	[−2.19, −2.05]	100.00	0.00	1.00	> 999
Intercept [3]	–1.63	[−1.70, −1.56]	100.00	0.00	1.00	> 999
Intercept [4]	–1.12	[−1.19, −1.06]	100.00	0.00	1.00	> 999
Intercept [5]	–0.50	[−0.56, −0.43]	100.00	0.01	1.00	> 999
Intercept [6]	0.12	[0.05, 0.18]	99.75	35.01	1.00	0.90
Intercept [7]	0.87	[0.80, 0.93]	100.00	0.00	1.00	> 999
Intercept [8]	1.72	[1.65, 1.79]	100.00	0.00	1.00	> 999
Intercept [9]	2.54	[2.47, 2.62]	100.00	0.00	1.00	> 999
Condition [Wanted]	0.05	[−0.04, 0.14]	82.66	80.28	1.01	0.02
Original [Good]	–0.58	[−0.61, −0.55]	100.00	0.00	1.00	> 999
Original [Mixed]	–1.04	[−1.07, −1.01]	100.00	0.00	1.00	> 999
Condition [Wanted]: Profile [Good]	–0.06	[−0.09, −0.02]	98.85	96.53	1.00	0.06
Condition [Wanted]: Profile [Mixed]	–0.10	[−0.14, −0.06]	100.00	52.07	1.00	15.96

*Due to multicollinearity between thresholds, ROPE estimates may be unreliable.*

**FIGURE 14:**
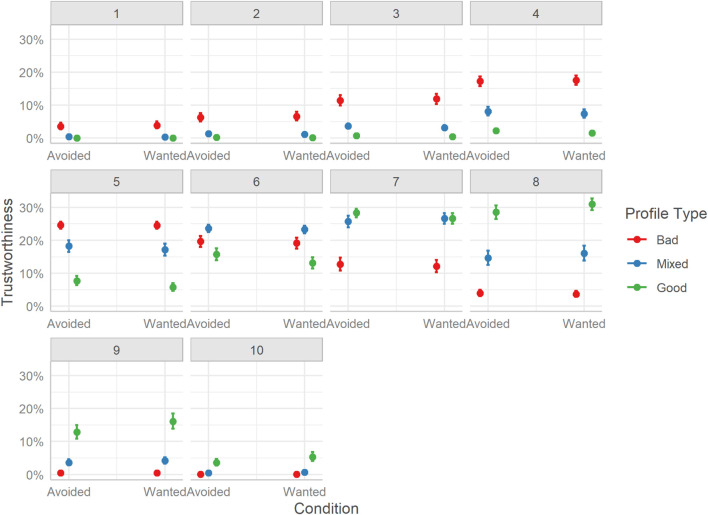
Marginal effects of trustworthiness. The posterior mean estimates of the probability of responses in each of the three Profiles (Bad, Good, Mixed) by the two experimental Conditions (Avoided or Wanted). Error bars indicate 95% credible intervals.

The data finds, as with sociability, no evidence for a difference in overall ratings between the two TRI groups, yet some (equivocal) evidence for an interaction effect with profile type. This potential interaction was probed and plotted in Figure 14.

Considering differences between the profile types, by TRI group, pairwise contrasts reveal that, overall, hosts of Bad profiles were rated as less trustworthy than hosts of Mixed profiles (Avoided, BF_10_ > 999; Wanted, BF_10_ > 999), or Good profiles (Avoided, BF_10_ > 999; Wanted, BF_10_ > 999). Unlike Study 1, hosts of Good profiles were rated more trustworthy than hosts of Mixed profiles (Avoided, BF_10_ > 999; Wanted, BF_10_ > 999). Considering differences between TRI groups by profile type, the data suggest no difference in ratings of Bad profiles, Mdn = 0.04, HDI_89__%_ [−0.09, 0.17], BF_10_ = 0.10, or Mixed profiles, Mdn = −0.08, HDI_89__%_ [−0.21, 0.06], BF_10_ = 0.01. A very small difference between ratings of Good profiles is suggested by the data, with the Wanted TRI group rating hosts as more trustworthy than the Avoided TRI group, Mdn = −0.19, HDI_89__%_ [−0.32, −0.05], BF_10_ = 0.11, yet the uncertainty around the estimate is quite large and the BF supports more the absence of a difference.

Finally, considering the distribution of ratings, we see that Bad profiles had a higher probability of receiving low trustworthiness scores (i.e., 1-4) and a low probability for high scores (i.e., 7-10). Mixed profiles had a more Gaussian-like distribution of scores, particularly favoring the middle of the scale (i.e., 5-7). Good profiles had a low probability of being rated using the lower-end of the scale, and a much higher probability of having ratings of 8, 9, and 10; however, the top-end of the scale seems to be rarely used.

##### Credibility

The model considering the effects of Condition and Profile on users’ ratings of the credibility of the information provided by hosts is reported in Table 15.

**TABLE 15:** Summary of the model fitted to the credibility ratings, considering the experimental condition (Avoided or Wanted) and Profile type (Good, Mixed, and Bad).

	β	89% HDI	PD	% in ROPE	*R̂*	BF_10_
Intercept [1]	–2.76	[−2.85, −2.67]	100.00	0.00	1.00	> 999
Intercept [2]	–2.32	[−2.40, −2.23]	100.00	0.00	1.00	> 999
Intercept [3]	–1.90	[−1.98, −1.82]	100.00	0.00	1.00	> 999
Intercept [4]	–1.41	[−1.49, −1.33]	100.00	0.00	1.00	> 999
Intercept [5]	–0.86	[−0.94, −0.78]	100.00	0.01	1.00	> 999
Intercept [6]	–0.25	[−0.32, −0.17]	100.00	0.10	1.00	> 999
Intercept [7]	0.54	[0.46, 0.62]	100.00	0.00	1.00	> 999
Intercept [8]	1.47	[1.39, 1.55]	100.00	0.00	1.00	> 999
Intercept [9]	2.34	[2.25, 2.43]	100.00	0.00	1.00	> 999
Condition [Wanted]	0.05	[−0.05, 0.16]	79.43	75.20	1.00	0.18
Original [Good]	–0.47	[−0.50, −0.44]	100.00	0.00	1.00	> 999
Original [Mixed]	–0.77	[−0.80, −0.74]	100.00	0.00	1.00	> 999
Condition [Wanted]: Profile [Good]	–0.10	[−0.14, −0.06]	100.00	46.29	1.00	15.01
Condition [Wanted]: Profile [Mixed]	–0.08	[−0.12, −0.04]	99.89	84.07	1.00	0.57

*Due to multicollinearity between thresholds, ROPE estimates may be unreliable.*

**FIGURE 15:**
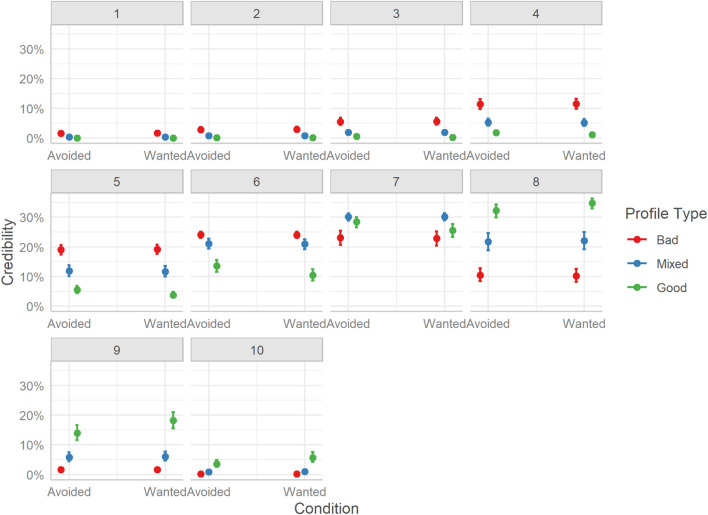
Marginal effects of trustworthiness. The posterior mean estimates of the probability of responses in each of the three Profiles (Bad, Good, Mixed) by the two experimental Conditions (Avoided or Wanted). Error bars indicate 95% credible intervals.

The model reveals that credibility ratings were unaffected by TRI group but were affected by profile type. Again, the model indicates a potential interaction that requires further consideration. See Figure 15.

The pairwise contrasts for profile types by TRI group revealed a near-identical pattern to that of trustworthiness, with Bad profiles being rated as containing less credible information than both Mixed (Avoided, BF_10_ > 999; Wanted, BF_10_ > 999) and Good (Avoided, BF_10_ > 999; Wanted, BF_10_ > 999). The information on Good profiles was rated as more credible than for Mixed profiles (Avoided, BF_10_ > 999; Wanted, BF_10_ > 999).

Considering differences between TRI group by profile type, a similar pattern to trustworthiness emerged, with no evidence for rating differences between Avoided and Wanted for either Bad (Mdn = 0.01, HDI_89__%_ [−0.14, 0.16], BF_10_ = 0.01), Mixed (Mdn = −0.02, HDI_89__%_ [−0.17, 0.14], BF_10_ = 0.01), or Good profiles (Mdn = −0.22, HDI_89__%_ [−0.38, −0.07], BF_10_ = 0.15), although the latter did show a trend for higher ratings in the Wanted condition.

Considering the distribution of ratings, Bad profiles had a higher probability of reaching low credibility ratings, but also more moderate ratings and almost no top-end ratings. Mixed profiles received predominantly moderate ratings, while Good profiles had higher probabilities for being rated with the top-end of the credibility scale.

##### Relationship Between Measures

As in Study 1, there was little evidence to suggest the experimental condition (here, Avoided or Wanted) impacted users’ perceptions of hosts. Thus, to verify the integrity and replicability of the findings reported in Study 1, we ran correlations between the three perception measures across the three profile types. See Table 16.

**TABLE 16:** Correlations Between Perception of Hosts Measures for Each Profile Condition.

Profile		Kendall’s Tau	BF_10_	*p*-value	89% HDI	
***Bad***						

Sociability	- Trustworthiness	0.65***, + + +	> 999	< 0.001	[0.59	0.70]
Sociability	- Credibility	0.33***, + + +	> 999	< 0.001	[0.27	0.39]
Credibility	-Trustworthiness	0.48***, + + +	> 999	< 0.001	[0.42	0.53]

***Mixed***						

Sociability	- Trustworthiness	0.62***, + + +	> 999	< 0.001	[0.55	0.67]
Sociability	- Credibility	0.40***, + + +	> 999	< 0.001	[0.34	0.45]
Credibility	-Trustworthiness	0.59***, + + +	> 999	< 0.001	[0.52	0.64]

***Good***						

Sociability	- Trustworthiness	0.62***, + + +	> 999	< 0.001	[0.56	0.67]
Sociability	- Credibility	0.52***, + + +	> 999	< 0.001	[0.45	0.57]
Credibility	-Trustworthiness	0.72***, + + +	> 999	< 0.001	[0.67	0.77]

* p < 0.05, **p < 0.01, ***p < 0.001; + BF10 > 10, ++ BF10 > 30, +++ BF10 > 100; N = 354.

Study 2’s data reveals a similar pattern of relationships. Across all profile types, the three host perception measures indicate strong positive correlations in ratings. While all correlations show strong relationships, the link between sociability and credibility seems, across all profile types, to be the lowest, followed by credibility and trustworthiness, while the strongest relationship is the one between sociability and trustworthiness. Indeed, investigating the difference in correlations (see Supplementary Material) revealed that for Bad profiles S-T is larger than in both C-T and C-S. For Mixed profiles, S-T is not found to be significantly different from C-T but is larger than C-S. For Good profiles, S-T, surprisingly, is smaller than C-T but larger than C-S. This may indicate that as profile quality goes down the relationship between host’s perceived characteristics and information credibility decreases, while when profile quality is high (i.e., Good profiles) credibility is more equivalent to host trustworthiness than it is with host sociability.

### Discussion

Study 2 supports the predictions that users have the ability to judge the diagnosticity of TRI elements on SE platforms and that the type of TRI elements presented can impact rental decisions. Displaying profiles with pairs of Wanted TRI led to more rent decisions for Good profiles and fewer for Bad profiles, compared to seeing Avoided TRI pairs (**H_6_**). Mixed profiles were rented in similar amounts in both experimental groups. Speculatively, mixed quality profiles may be harder to identify, or cannot be influenced by the type of TRI. This effect can be resolved by considering the accuracy data, which displayed an overall effect on accuracy with the Wanted group (70%) being more accurate than the Avoided group (65%). Thus, users seeing Wanted TRIs were more likely to identify that a profile was Good, Mixed, or Bad, indicating that diagnosticity varies across TRI elements (**H_5_**). Looking at the Mixed profiles, given that the Wanted group was more accurate in their classifications than the Avoided group, together with the rent decision data indicated a plateau for rental of mixed quality profile rooms (∼ 44%). Namely, even if users were more accurate in detecting that a profile was of mixed quality their decision to rent was the same.

Considering judgment bias (**H_8_**), Wanted TRIs led to better classification of profiles compared to Avoided TRIs, especially for Good and Mixed profiles. The response bias pattern was similar in both groups, with the only difference being in the magnitude of the errors, not direction. Judgment confidence (**H_7_**) was not affected by group and showed the same pattern for the profile conditions as in Study 1 (i.e., Good and Bad being judged at similar confidence levels, and higher than Mixed).

With respect to the host perception measures (**H_9_**), there were no differences between the Wanted and Avoided groups in terms of sociability, trustworthiness, or credibility; although a trend for Good profiles being perceived as more trustworthy and credible in the Wanted than the Avoided group was indicated by the data. Unlike in Study 1, the pattern in ratings matched the quality of the profiles (**H_10_**), as Good profiles were rated more highly on all metrics than were Mixed profiles, which in turn were rated more highly than Bad profiles.

The different types of TRIs were not found to impact perceptions of hosts, and in contrast to Study 1, the ratings matched the quality of profiles, with the highest ratings for good profiles and the lowest for bad profiles. Given that in Study 2 there were two TRIs per profile instead of three or seven as in Study 1, one explanation for the clearer pattern of results may relate to cognitive load differences resulting from the difference in decision-making processes (Harries and Harvey, 2000; Gigerenzer and Gaissmaier, 2011). Here, participants neither had to first consider and select which elements they wanted to see (Study 1’s Reveal condition) nor incorporate information from three separate cues (Study 1’s Visible condition). This reduced task complexity may have permitted a clearer judgment pattern to emerge. Thus, the more simple and streamlined decision process may explain the more expected pattern of results, as users could more easily rely on more heuristic-based judgments (Tversky and Kahneman, 1974). This is also reflected in the correlations among measures, which mirror those of Study 1 but are more robust and precise due to the increased sample size. These indicate that users develop a homogenized view of hosts which results in similar impressions about them on several dimensions which fit their expectations (Nisbett and Wilson, 1977; Ariely et al., 2003). This is in line with investigations reporting that in general SE populations seem to be quite similar and predictable (e.g., Koh et al., 2019). Moreover, this may also provide a more rational explanation to some of the overly positive opinions typically found on SE platforms (Zervas et al., 2021), as users may have limited information to form adequate impressions and rely on more generic (and consistent) impressions of others (see Nicolau et al., 2020).

## Conclusion

The SE is a novel and ever-developing environment where decisions center around P2P interactions, relying on TRI provided by other community members (i.e., UGC). Given the added risks and uncertainty associated with such markets, we investigated how accurate and/or biased users are when making decisions about the quality of accommodation-style profiles using TRI. Across two experimental studies, the data indicate that users are fairly accurate in their assessment of the quality of profiles but, when making errors tend to overestimate quality. Additionally, users seem capable of distinguishing the diagnostic value of various TRI elements (Study 1), and when provided with either the most frequently selected combinations of TRI elements or the most avoided, a difference in accuracy and judgment is observed (Study 2).

Study 1 explored the effect of TRI elements on judgment and decision-making, considering how varying the number of elements and users’ ability to select information would impact accuracy, bias, and perception. Users made similar decisions when shown either a full profile with seven TRI elements or a profile containing three user-selected TRI elements. Additionally, while users displayed high accuracy, they were also biased toward overestimating quality, in line with the findings in Zloteanu et al. (2018). Study 2 investigated the diagnosticity of user-selected TRIs. Presenting users with pairs of TRIs that were (in)frequently selected in Study 1 to uncover if different TRIs carry different levels of diagnosticity for user decision-making. The data showed that, indeed, showing users “wanted” TRIs led to higher accuracy, increased rent decisions for good profiles, and lowered rent decisions for bad profiles, while mixed profiles rentals stayed constant. However, confidence was similar and generally high among both TRI conditions, which may suggest this added diagnosticity was occurring without conscious awareness. As in Study 1, users showed an upward bias in misclassification. Taken together this may indicate that a change in perception and judgment may occur between incorporating two or three elements into one’s decision-making. Thus, while SE users may require around three diagnostic TRI elements to make informed judgments, the higher cognitive load leads to additional, potentially less conscious, factors, such as comfort and homeliness, to have a role in the decision-making process.

Overall, the main hypothesis of our research was supported. Users presented with diagnostic TRI elements in a SE-like environment can produce accurate judgments relating to the quality of the services or products offered. Furthermore, we found support for the upward bias in responses, such that when users misclassified quality they were more likely to rate it in a higher tier than in a lower one. Study 1 also demonstrated that users can reach peak accuracy relying on only three TRI elements, providing support for our information discounting explanation (Zloteanu et al., 2018), while Study 2 provided support for the users’ ability to identify the most diagnostic elements to aid their decision-making. Overall, it seems that showing users only two TRI elements leads to a more predictable pattern of results (i.e., Good > Mixed > Bad).

In addition to accuracy, our data indicate that judgment confidence tended to be quite high for all decisions users made, suggesting that even in an uncertain environment like the SE (Phua, 2019; Luo et al., 2021) people are confident in their choices. The high correlations between the three host metrics indicate that users tend to have a halo-like judgment of individuals on SE platforms (Nicolau et al., 2020). Given the considerable lack of negative feedback in real-world SE platforms (Zaki et al., 2021), a caveat of this is that, currently, the diagnosticity present in our artificial platform may be higher and more apparent than in SE platforms, at least from a distributional perspective (see Koh et al., 2019).

### Theoretical Implications

The current findings have relevant implications to our understanding of human judgment in SE environments as well as recommendations for how such platforms can evolve to ensure transparent and informed decision-making for its users.

The main finding of the research, and in support of our primary hypothesis, is that people are accurate at detecting the quality of SE accommodation profiles if they are presented with diagnostic TRI. Implicitly, this raises the importance of investigating in detail the usefulness of information on real-world SE platforms and the need to understand how users reach their decisions. At present, the faux prosocial culture that is being cultivated seems to be at odds with our suggestion, as more emphasis is placed on fostering a “5-for-5” mentality in interactions than on ensuring people have the information necessary to make accurate judgments (Livan et al., 2017; Zervas et al., 2021); this is especially evident given the rise in systems which encourage users to provide feedback in exchange for compensation (e.g., reward-for-feedback; Li et al., 2020). Additionally, it is clear that not all TRI elements carry the same diagnostic value, at least for user decision-making.

The results from the mixed profile condition may have relevant implications for our understanding of user cognition in SE environments. The effect on perceptions of host trustworthiness, sociability, and credibility in Study 1 compared to Study 2 indicate a potential cognitive strategy and decision bias. Research finds that people over-acquire information when they consider data to be “noisier” (Harvey and Bolger, 2001), thus one would assume that differences would have emerged in Study 1 between the Visible and Reveal conditions for the mixed profiles, as users had access to more TRIs in the former group. Assuming that not all users in the Reveal condition selected optimal TRIs, while all users in the Visible condition had access to these optimal cues it would stand to reason that a difference would emerge in classification accuracy. Yet, no differences were found (although the data does indicate such a trend), while a difference is seen when compared to Study 2. Our cognitive load explanation can account for this, as although the Visible condition did contain more optimal TRI, the benefit of its presence may have been outdone by the increased complexity of the decision (i.e., optimally integrating all seven TRI elements). Thus, when investigating the decision-making strategies of users, research must consider decision complexity and ease of cue retrieval (Harries and Harvey, 2000; Gigerenzer and Gaissmaier, 2011).

To explain the more linear findings in Study 2, we posit that two TRI elements were insufficient for users to make informed judgments regarding the hosts leading to them defaulting to pre-existing patterns of judgment (Kahneman and Tversky, 1973). Specifically, if we treat Study 1’s data as reflecting realistic user beliefs, then in Study 2 users made inferences based on their rent and accuracy decisions to inform their host perceptions judgments as TRI was insufficient or too uncertain (although, the average judgment confidence in both studies was similar; Study 1, *M* = 7.54, *SD* = 1.54, and Study 2, *M* = 7.52, *SD* = 1.20). One of the central complaints users typically have on SE platforms relates to uncertainty and ambiguity (Phua, 2019; Luo et al., 2021). Thus, while two TRI elements may be sufficient for accuracy and rental judgments, more may be required to form complete inferences about hosts. We cannot speculate on which of the two components is more relevant for the SE environment, especially as research finds an increasing shift in people’s preferences from the informal mentality to a more business-like expectation of services (Bucher et al., 2018; Zhu et al., 2019) which is at odds with SE users still greatly valuing having an authentic local experience and the ability to create meaningful social bonds (Mody et al., 2019).

The generally high levels of rent decisions, confidence, and host perception ratings also speak to another component of the SE: people display a general high level of trust. The current pattern of results echoes past research on people’s perception of SE platforms (Livan et al., 2017; Zloteanu et al., 2018; Zaki et al., 2021). Importantly, trust seems to have a transitive property, as trust in users tends to result in trust in the platform (and vice-versa) (but see Yang et al., 2019). We consider this transitive property must be considered carefully, as it may stem from a false equivalence that if the platform is safe, so are the associated interactions (cf. Martínez-Navalón et al., 2021). Yet given the P2P nature of the SE, this may not be so (see Earle and Cvetkovich, 1999). Indeed, research suggests that other non-UGC plays a significant role in trust-building on such platforms (Kong et al., 2020). Understanding how perceptions of the platform versus perceptions of the community impact judgment would be relevant to understanding how trust is formed in SE marketplaces.

### Practical Implications

Our studies suggest that SE platforms would benefit from ensuring the information provided to users is accurate and diagnostic of quality, and to curtail the fostering of a hyper-positive and faux prosocial behavior. The results from our experiments have clear and important implications for platform owners/managers, policymakers, and future SE platform developments.

One clear application is in designing the platforms to achieve specific goals and promote desired behavior. In recent years, there has been a push toward making people engage with and adopt SE platforms that promote ethical designs (Saura et al., 2021a), sustainable economic development, and energy efficiency (Dabbous and Tarhini, 2021). Platform managers for such enterprises should ensure specific TRI is present on their platforms to maximize the likelihood that users will find them trustworthy and credible, increasing engagement and positive perceptions. Importantly, these can be used to foster different behaviors which relate to the sustainability of SE platforms, such as shared community values (Nadeem et al., 2021). Derivations of our current work can be applied to identify elements that can increase trust, a component that is critical to the sustainability and growth of an SE platform (Teubner, 2014). Moreover, proper platform design regarding TRI can assist in reducing uncertainty (e.g., from excessive information, confusion, and cognitive overload) which usually produce negative effects on user experience and engagement (Ding et al., 2021).

From the opposite perspective, tailoring the type of TRI and engagement mechanisms present on such platforms can be used to reduce non-beneficial behavior (e.g., the spread of non-diagnostic or biasing information). By extension, policymakers must carefully monitor the behavior of SE platforms in light of our findings to ensure that such tactics are not used in ways that disadvantage users or place them at higher risk (e.g., using specific TRI to inflate perceptions of safety and trust).

Although, presently, no specific pattern in TRI pairs emerged, Wanted pairs typically containing a combination of 1 trust element (e.g., reviews from guests or hosts) and 1 reputation element (e.g., star ratings, number of reviews) and no social media element, while Avoided pairs had a higher combination of two reputation elements and included the social media element. Speculatively, this would suggest that users seeing a trust + reputation element combination produced the most accurate inferences. Also, most wanted elements were associated with information generated endogenously on the platform (i.e., reviews and ratings) rather than exogenously (e.g., social media presence and cross-platform reputation). Future research could explore these combinations in more detail to understand why some are optimal. Importantly, this speaks to the issue of SE platforms encouraging, if not demanding, that people must share an ever-increasing amount of personal information under the assumption of it leading to more informed decision-making. Thus, SE operators must carefully consider what information is necessary and provide a balance between transparency and intrusiveness.

Prosumers (here, hosts) can also consider the relevance of our findings. Hosts may be able to make themselves more attractive to potential guests by ensuring the TRI which best encapsulates their quality of services and offered experience are present and highlighted. One’s digital identity – the information they present in an online space – is becoming more and more important, serving as an identifiable brand for the individual. Considering the vast competition on SE platforms, having a well-curated digital identity, which contains relevant TRI, can be essential to ensuring sustainability and survivability within this ecosystem.

From an efficiency and cost perspective, our findings illustrate that users do not require all TRI that is typically found on accommodation-style SE platforms. Here, our results mirror those of Zloteanu et al. (2018), whereby there is no appreciable difference in user judgment if presenting them with more than three TRI elements. As seen in Study 1, accuracy, bias, and judgment confidence were unaffected by seeing seven elements instead of three. Thus, platform managers and operators may utilize this information to streamline their platforms and reduce costs associated with collecting, storing, and presenting unnecessary information as well as anticipate which information users may request when attempting to make decisions (e.g., for automated customer service platforms; Saura et al., 2021b). Furthermore, such streamlining of SE platforms confers additional benefits relating to privacy and security. While in certain domains, large sources of personal information can be beneficial (e.g., public health reasons; Ribeiro-Navarrete et al., 2021), here, providing only the required level of TRI can ensure that users are not urged or expected to provide more information that they need to optimally participate in the SE ecosystem.

Lastly, taking the above information together, both policymakers and platform managers have clear resources they can employ to facilitate the adoption and use of SE platforms which provide a benefit to their populations. Understanding which TRI elements matter to users, how they can influence and improve decision-making, and encourage engagement and community formation can be useful in promoting the use of benevolent (e.g., NGOs; Saura et al., 2020), allowing them to compete (e.g., by streamlining information) with more established and larger competitors.

### Limitations and Future Directions

Given the novelty of this line of research, a few considerations and future directions are discussed.

An important caveat is that to ensure that our effects were due to the TRI manipulations we eliminated other sources of variability and uncertainty in our SE profiles. However, this also created a “best case” scenario for user performance. Thus, real-world data and effects may be less clear and much more varied, introducing other sources of bias and judgment preference (e.g., Edelman et al., 2017). That said, research has suggested that there is great homogeneity within userbases on such platforms (Koh et al., 2019). Nonetheless, the fact that users can be accurate if given the right circumstances does still provide valuable and novel insights into human decision-making within the SE ecosystem.

A caveat of our current paradigm is the potentially contentious terminology employed (i.e., good, mixed, bad). These categories may not reflect how users judge profiles, as they may consider quality on a spectrum (e.g., a “rentability” scale), or potentially in a binary fashion. The differences in rent decisions between the three profile types indicate the former more than the latter. Additionally, our Mixed condition was not a pure blend of good and bad TRI elements, but a separate “moderate” category where the information contained in the TRIs reflected this level in quality. If users were shown a blend of good and bad TRIs the pattern of results may look different.

A final limitation is the inability to unpack in more depth the various TRI pair combinations, especially with respect to users’ final perceptions and judgments. Replications of our work may use our results to estimate the pattern of effects, however, this would require a very large sample size to account for any differences in judgment patterns.

As the research presented offers a novel approach to investigating user judgment in SE environment, more work must build on our findings to understand the strategies and intricacies of user behavior. Here, we have found that different TRI elements not only carry different diagnostic value in determining SE profile quality, but that specific combinations result in better/worse decision-making. Future work may focus on the potential bias that specific TRI combinations can produce. Instead of providing users with diagnostic TRI elements, it would be possible to present TRI elements that lead to specific misclassifications (e.g., upward or downward bias), thereby investigating whether it is possible to sway people’s perceptions of quality. Additionally, researchers could consider the weight users assign to different TRI elements, either by comparing the judgment impact of each TRI or when presented with two conflicting TRIs (e.g., one positive and one negative). Such an exploration could uncover the type of strategy SE users adopt when making decisions (e.g., tallying or take-the-best; Bobadilla-Suarez and Love, 2018). Extensions must also consider monetary constraints on decision-making and TRI choice. Here, there were no costs to users misclassifying profiles or selecting non-diagnostic TRIs. In the real world, poor decisions on SE platforms can result in negative experiences and costly losses. Future work could introduce a monetary element and investigate the impact it has on TRI choice, judgment formation, and decision outcome.

Varying the non-TRI elements on SE profiles would be an alternative direction for this research. Our aim was to unpack the effects of TRI variance of judgment, meaning all non-TRI elements were kept constant to avoid confounds (e.g., all photos were of similar quality rooms). Factors such as property type, type of host, and other non-TRI elements may also impact user judgments (Xu, 2020; Ding et al., 2021). Similarly, our studies focus on the initial stage of an SE interaction, but research finds that perceptions of trust can shift post-interaction (Phua, 2019). Tracking the changes in perception and judgment at multiple points in an SE interaction would uncover which stage carries the most impact. Thus, the interdynamics of users on SE platforms must be considered in more detail.

Our focus was on guest judgment toward hosts, yet the other side of the equation is just as important: how do hosts behave in SE environments? Hosts themselves can perceive and judge different types of guests based on their profiles and their respective digital identities (Zloteanu et al., 2018; Zhang et al., 2021). Similarly, research has found that the type of properties offered by different hosts can lead to different judgments from users, especially regarding intentions to interact and/or revisit (Ding et al., 2021). To properly understand user behavior in the SE, one needs to consider the complete dynamics of the system, attempting to reach a causal and integrated model.

Lastly, considerations should be given to how SE interactions and functioning has been impacted by recent events (e.g., the COVID-19 pandemic; Jang et al., 2021). Engagement with SE platforms is clearly impacted not only by internal forces but by external ones as well. Such elements undoubtedly play a significant role in how people view and desire to use SE services, as well as the factors they deem most pertinent to their decisions.

## Data Availability Statement

The raw data supporting the conclusions of this article will be made available by the authors, without undue reservation.

## Ethics Statement

The studies involving human participants were reviewed and approved by University College London Research Ethics Committee (CEHP/2015/534). The patients/participants provided their written informed consent to participate in this study.

## Author Contributions

MZ, NH, DT, and GL: conceptualization and writing–review and editing. MZ: data curation, formal analysis, investigation, and methodology. GL: funding acquisition, project administration, and supervision. MZ and GL: writing–original draft. All authors contributed to the article and approved the submitted version.

## Conflict of Interest

The authors declare that the research was conducted in the absence of any commercial or financial relationships that could be construed as a potential conflict of interest.

## Publisher’s Note

All claims expressed in this article are solely those of the authors and do not necessarily represent those of their affiliated organizations, or those of the publisher, the editors and the reviewers. Any product that may be evaluated in this article, or claim that may be made by its manufacturer, is not guaranteed or endorsed by the publisher.

## Footnotes

1
www.gorilla.sc

2 The PD metric can be converted into a frequentist *p*-value (Makowski et al., 2019a); for example, a PD = 96.96% is equivalent to a *p* = 0.061
