# Supplementary material for: Judgments in the Sharing Economy: The Effect of User-Generated Trust and Reputation Information on Decision-Making Accuracy and Bias
Source: Front Psychol. 2021 Nov 16;12:776999. doi: 10.3389/fpsyg.2021.776999 (PMC8637778; doi:10.3389/fpsyg.2021.776999)
Supplement: Supplementary file 1 [file Data_Sheet_1.docx]

Supplementary Material

# TRI generation process

We provide additional details on the creation of the three profile quality types: Good, Mixed, and Bad. In the below table we present the element variance for each TRI element for each respective profile condition. A profile in each of the three conditions was generated based on a set of underlying criteria to ensure consistency and sufficient difference to allow users to determine the quality of the information presented.

| **TRI Element** | **Range of values** | | |
| --- | --- | --- | --- |
|  | **Good** | **Mixed** | **Bad** |
| 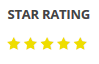 | Number of stars = 4, 4.5, 5 | Number of stars = 3.5, 4, 4.5 | Number of stars = 2.5, 3, 3.5 |
| 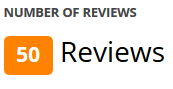 | Min = 75, Range = 25 | Min = 25, Range = 50 | Min = 1, Range = 24 |
| 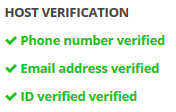 | Number of ticked/green items = 3 (full list) | Number of ticked/green items = 2 or 3 | Number of ticked/green items = 1 or 2 |
| 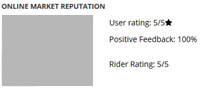 | Ebay, Min feedback = 86%, Max = 100%; Ratings range = 4.5-5 (0.1 increments)  Uber, Ratings range = 4.5-5 (0.1 increments); | Ebay, Min feedback = 73%, Max = 87%; Ratings range = 4-4.5 (0.1 increments)  Uber, Ratings range = 4-4.5 (0.1 increments) | Ebay, Min feedback = 60%, Max = 74%; Ratings range = 3-3.5 (0.1 increments)  Uber, Ratings range = 3-3.5 (0.1 increments) |
| 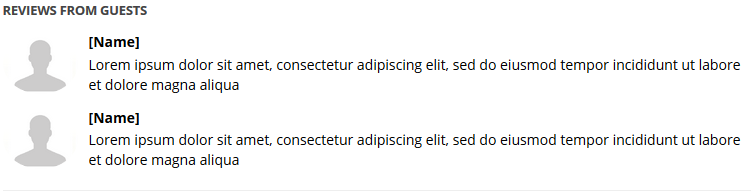 | 3x Reviews, randomly selected in a combination of 2x Good + 1x Mixed | 3x Reviews, randomly selected in a combination of 2x Mixed + (either) 1x Good/Bad | 3x Reviews randomly selected in a combination of 2x Bad + 1x Mixed |
| 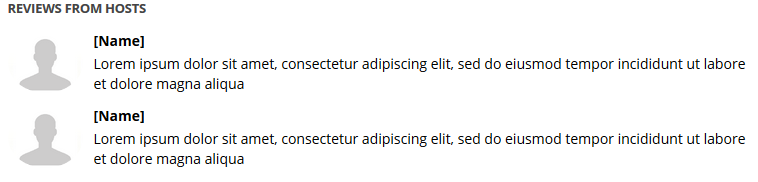 | 3x Reviews, randomly selected in a combination of 2x Good + 1x Mixed | 3x Review, randomly selected in a combination of 2x Mixed + (either) 1x Good/Bad | 3x Reviews randomly selected in a combination of 2x Bad + 1x Mixed |
| 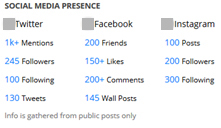 | Instagram, Posts, Min = 550, Range = ± 10-100; Followers, Min = 1k, Range = ±1-3k; Following, Min = 1k, Range = ±1-6k;  Twitter, Mentions, Min = 1k, Range = ±0.1-0.3k; Followers, Min = 200, Range = ±10-50; Following, Min = 300, Range = ±50-200; Tweets, Min = 300, Range = ±10-100;  Facebook, Friends Min = 300, Range = ±100-400; Likes, Min = 1k, Range = ±0.1-0.5k; Comments, Min = 400, Range = ±100-300; Wall Posts, Min = 150, Range = ±10-50 | Instagram, Posts Min = 200, Range = ± 10-100; Followers, Min = 1k, Range = ±1-3k; Following, Min = 600, Range = ±100-400;  Twitter, Mentions, Min = 100, Range = ±0.1-0.9k; Followers, Min = 150, Range = ±10-50; Following, Min = 250, Range = ±10-90; Tweets, Min = 200, Range = ±10-100;  Facebook, Friends Min = 200, Range = ±50-150; Likes, Min = 100, Range = ±0.1-0.9k; Comments, Min = 150, Range = ±50-300; Wall Posts, Min = 110, Range = ±10-50 | Instagram, Posts Min = 150, Range = ± 10-80; Followers, Min = 200, Range = ±10-100; Following, Min = 400, Range = ±50-200;  Twitter, Mentions, Min = 40, Range = ±5-20; Followers, Min = 100, Range = ±10-100; Following, Min = 200, Range = ±50-200; Tweets, Min = 150, Range = ±10-100;  Facebook, Friends Min = 3150, Range = ±10-100; Likes, Min = 80, Range = ±10-40; Comments, Min = 120, Range = ±10-40; Wall Posts, Min = 80, Range = ±10-40 |

*Note.* Commercial logos have been removed for publication.

# Artificial accommodation-style platform

We provide a brief overview of the non-TRI elements which were present on each profile. These were selected and produced to mirror genuine SE platforms, ensuring that users would not require much time to familiarize themselves with how the platform functions or its layout. Below is a description of each element comprising the profiles of hosts shown to participants, along with an example of how it appeared in the experiments. For more details see Zloteanu et al. 2018.

## Room photos


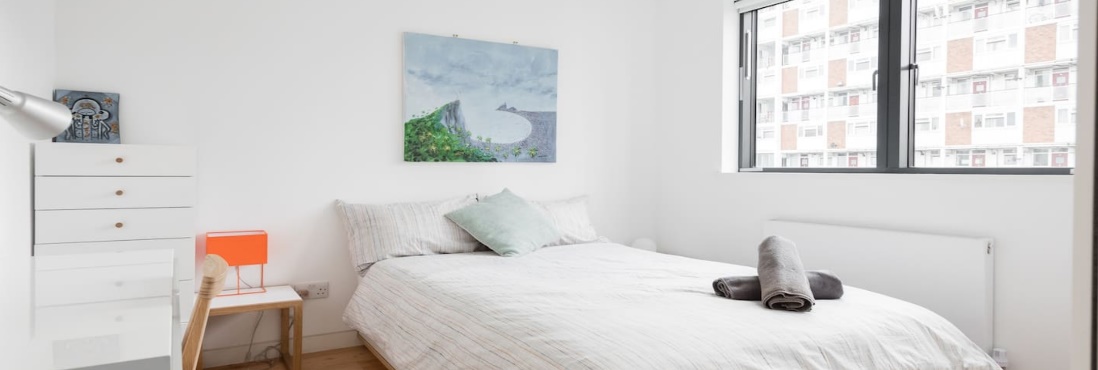


The images of the rooms were scrapped from Airbnb based on specific conditions. All images were from a single borough in the Greater London area and were within 1 SD of the average room price, offering similar amenities and overall quality. 30 such images were used.

## Titles


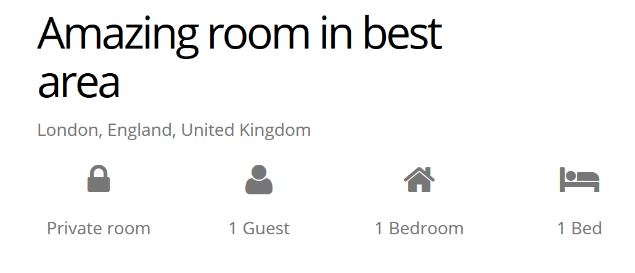


Tiles were generated based on similar descriptions of the rooms used for the images above. The names were created to avoid identifying or specific information and had a length of 3-7 words.

## Descriptions of room


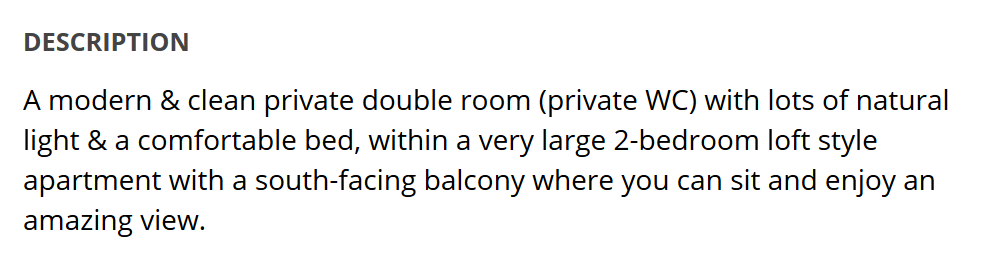


The room descriptions were generated with information typically seen for the rooms selected for photos and titles. Alterations were made to eliminate location or subjective information and were constrained to 20-50 words.

## Profile photos


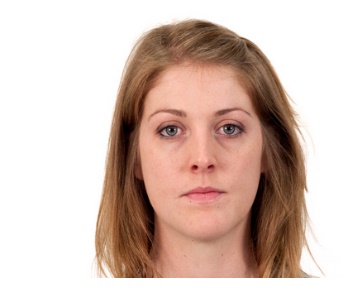


All profile photos - for the Host profile and the User reviews – were generated using the Chicago Face Database (Ma et al., 2015). The database has ratings on several key facial features, including facial dominance, trustworthiness, and attractiveness (±1 SD from the mean on each characteristic). These factors were controlled for when selecting the stimuli used in the profile generation. For the host profiles photos only, the ethnicity was kept constant (White-Caucasian).

# Study 1: Differences between correlations

To explore users’ judgments, we considered if there were statistically significant differences in the magnitude of correlations among the three host perception metrics: sociability (S), trustworthiness (T), and credibility (C). Although, as reported in the Main text, all correlations were positive and moderate-to-large, this does not preclude the fact that certain relationships may be more/less strongly correlated. To investigate such differences, we ran Steiger’s Z tests considering both differences in the strength of correlations between profile conditions (Good, Mixed, Bad) and differences within. Interpretations are provided in the Main text.

## Sociability – Trustworthiness

Bad-Mixed: Steiger’s Z = 0.20, *p* = .843, *r_diff_* = 0.01, 95% CI [-0.11, 0.13], *ns*.

Bad-Good: Steiger’s Z = -0.95, *p* = .344, *r_diff_* = -0.06, 95% CI [-0.17, 0.06], *ns*.

Mixed-Good: Steiger’s Z = -1.23, *p* = .219, *r_diff_* = -0.07, 95% CI [-0.18, 0.04], *ns*.

## Sociability – Credibility

Bad-Mixed: Steiger’s Z = -1.64, *p* = .102, *r_diff_* = -0.16, 95% CI [-0.36, 0.03], *ns*.

Bad-Good: Steiger’s Z = -3.62, *p* < .001, *r_diff_* = -0.39, 95% CI [-0.58, -0.17], *significant.*

Mixed-Good: Steiger’s Z = -2.51, *p* = .012, *r_diff_* = -0.21, 95% CI [-0.39, -0.05], *significant*.

## Trustworthiness – Credibility

Bad-Mixed: Steiger’s Z = -1.90, *p* = .057, *r_diff_* = -0.18, 95% CI [-0.36, 0.01], *ns*.

Bad-Good: Steiger’s Z = -4.02, *p* < .001, *r_diff_* = -0.38, 95% CI [-0.56, -0.19], *significant*.

Mixed-Good: Steiger’s Z = -2.55, *p* = .011, *r_diff_* = -0.20, 95% CI [-0.36, -0.05], *significant*.

## Pairwise correlation comparisons – Bad profile condition

S-T vs. C-T: Steiger’s Z = 4.54, *p* < .001, *r_diff_* = 0.40, 95% CI [0.22, 0.58], *significant*.

S-T vs. C-S: Steiger’s Z = 5.71, *p* < .001, *r_diff_* = 0.49, 95% CI [0.32, 0.67], *significant*.

## Pairwise correlation comparisons – Mixed profile condition

S-T vs. C-T: Steiger’s Z = 2.86, *p* = .004, *r_diff_* = 0.21, 95% CI [0.07, 0.36], *significant*.

S-T vs. C-S: Steiger’s Z = 4.39, *p* < .001, *r_diff_* = 0.31, 95% CI [0.17, 0.47], *significant*.

## Pairwise correlation comparisons – Good profile condition

S-T vs. C-T: Steiger’s Z = 1.59, *p* = .111, *r_diff_* = 0.08, 95% CI [-0.02, 0.18], *ns*.

S-T vs. C-S: Steiger’s Z = 3.49, *p* < .001, *r_diff_* = 0.17, 95% CI [0.07, 0.29], *significant.*

# Study 2

## Pair selection process

The selection of the Avoided and Wanted pairs was done based on a statistical analysis of Study 1’s participants’ selection patterns. We tested the co-occurrences of certain selected items against a null hypothesis of random selection (encoded as a hypergeometric distribution, and multiple hypotheses testing Bonferroni-corrected 1% significance level). The analysis was run both in the case of pairs (i.e., how frequently certain pairs of TRI elements were co-selected as part of a triplet in the Reveal condition of Study 1, regardless of the third TRI element selected together with them) and at the level of full triplets.

The analysis revealed 6 pairs and 6 triplets to be overrepresented (i.e., “wanted"), and 12 pairs and 20 triplets to be underrepresented (i.e., “avoided”). Due to the very high number of “avoided” triplets, we chose to run study 2 with pairs, and we restricted the number of “avoided” pairs in the study from 12 to 6 by removing the 6 pairs of the type “host verification + *another TRI element*”, which were all underrepresented but still associated with relatively higher selection frequencies.

## Differences between correlations

As with Study 1, we explored differences in the magnitude of correlation pairs. Here, the focus was on differences in sociability, trustworthiness, and credibility correlations between the three profile conditions.

### Pairwise correlation comparisons – Bad profile condition

S-T vs. C-T: Steiger’s Z = 3.67, *p* < .001, *r_diff_* = 0.17, 95% CI [0.08, 0.26], *significant*.

S-T vs. C-S: Steiger’s Z = 7.08, *p* < .001, *r_diff_* = 0.31, 95% CI [0.23, 0.40], *significant*.

### Pairwise correlation comparisons – Mixed profile condition

S-T vs. C-T: Steiger’s Z = 0.73, *p* = .467, *r_diff_* = 0.03, 95% CI [-0.05, 0.12], *ns*.

S-T vs. C-S: Steiger’s Z = 5.40, *p* < .001, *r_diff_* = 0.22, 95% CI [0.14, 0.30], *significant*.

### Pairwise correlation comparisons – Good profile condition

S-T vs. C-T: Steiger’s Z = -2.83, *p* = .005, *r_diff_* = -0.10, 95% CI [-0.17, -0.03], *significant*.

S-T vs. C-S: Steiger’s Z = 3.38, *p* < .001, *r_diff_* = 0.11, 95% CI [0.05, 0.17], *significant.*

# References

Edelman, B. G., and Luca, M. (2014). Digital discrimination: The case of Airbnb. com. *Harvard Business School NOM Unit* Working Paper no, 14–054.

Edelman, B., Luca, M., and Svirsky, D. (2017). Racial Discrimination in the Sharing Economy: Evidence from a Field Experiment. *American Economic Journal: Applied Economics* 9, 1–22. doi:10/gf2q4d.

Ma, D. S., Correll, J., and Wittenbrink, B. (2015). The Chicago face database: A free stimulus set of faces and norming data. *Behav Res* 47, 1122–1135. doi:10/f7zbwd.

Zloteanu, M., Harvey, N., Tuckett, D., and Livan, G. (2018). Digital Identity: The effect of trust and reputation information on user judgement in the Sharing Economy. *PLOS ONE* 13, e0209071. doi:10/ggrjrd.
